# Alterations in chromosomal genes *nfsA*, *nfsB*, and *ribE* are associated with nitrofurantoin resistance in *Escherichia coli* from the United Kingdom

**DOI:** 10.1099/mgen.0.000702

**Published:** 2021-12-03

**Authors:** Yu Wan, Ewurabena Mills, Rhoda C.Y. Leung, Ana Vieira, Xiangyun Zhi, Nicholas J. Croucher, Neil Woodford, Elita Jauneikaite, Matthew J. Ellington, Shiranee Sriskandan

**Affiliations:** ^1^​ NIHR Health Protection Research Unit in Healthcare Associated Infections and Antimicrobial Resistance, Department of Infectious Disease, Imperial College London, London, United Kingdom; ^2^​ MRC Centre for Molecular Bacteriology and Infection, Imperial College London, London, United Kingdom; ^3^​ Department of Infectious Disease Epidemiology, School of Public Health, Imperial College London, London, United Kingdom; ^4^​ MRC Centre for Global Infectious Disease Analysis, School of Public Health, Imperial College London, London, United Kingdom; ^5^​ Antimicrobial Resistance and Healthcare Associated Infections Reference Unit, National Infection Service, Public Health England, Colindale, London, United Kingdom; ^†^​ Present address: Department of Microbiology, Queen Mary Hospital, Hong Kong S.A.R., PR China

**Keywords:** *Escherichia coli*, Nitrofurantoin, antimicrobial resistance, resistance mechanisms, chromosomal mutations, Predicting resistance

## Abstract

Antimicrobial resistance in enteric or urinary *Escherichia coli* is a risk factor for invasive *E. coli* infections. Due to widespread trimethoprim resistance amongst urinary *E. coli* and increased bacteraemia incidence, a national recommendation to prescribe nitrofurantoin for uncomplicated urinary tract infection was made in 2014. Nitrofurantoin resistance is reported in <6% urinary *E. coli* isolates in the UK, however, mechanisms underpinning nitrofurantoin resistance in these isolates remain unknown. This study aimed to identify the genetic basis of nitrofurantoin resistance in urinary *E. coli* isolates collected from north west London and then elucidate resistance-associated genetic alterations in available UK *E. coli* genomes. As a result, an algorithm was developed to predict nitrofurantoin susceptibility. Deleterious mutations and gene-inactivating insertion sequences in chromosomal nitroreductase genes *nfsA* and/or *nfsB* were identified in genomes of nine confirmed nitrofurantoin-resistant urinary *E. coli* isolates and additional 11 *E. coli* isolates that were highlighted by the prediction algorithm and subsequently validated to be nitrofurantoin-resistant. Eight categories of allelic changes in *nfsA*, *nfsB*, and the associated gene *ribE* were detected in 12412 *E. coli* genomes from the UK. Evolutionary analysis of these three genes revealed homoplasic mutations and explained the previously reported order of stepwise mutations. The mobile gene complex *oqxAB*, which is associated with reduced nitrofurantoin susceptibility, was identified in only one of the 12412 genomes. In conclusion, mutations and insertion sequences in *nfsA* and *nfsB* were leading causes of nitrofurantoin resistance in UK *E. coli *. As nitrofurantoin exposure increases in human populations, the prevalence of nitrofurantoin resistance in carriage *E. coli* isolates and those from urinary and bloodstream infections should be monitored.

## Data Summary

Sequence reads and genome assemblies of *E. coli* isolates IN01–09 have been deposited in the European Nucleotide Archive (ENA) under BioProject accession number PRJEB38850. Nucleotide and protein sequences of *nfsA, nfsB*, and *ribE* alleles in genomes of these nine isolates can be accessed in our database NITREc (github.com/wanyuac/NITREc). The database also offers nucleotide sequences of genetic elements related to the interrupted *nfsA/nfsB* regions in IN01–03. All supplementary files are available on figshare (https://doi.org/10.6084/m9.figshare.16638457.v1)[106].

**Impact Statement**

This study expands knowledge on the genetic basis of nitrofurantoin resistance in *E. coli* using whole-genome sequencing and in-depth genomic analysis. We report novel and previously discovered deleterious mutations in chromosomal genes *nfsA*, *nfsB*, and *ribE* as well as the interruption of *nfsA* and *nfsB* by insertion sequences, recapitulating the roles of oxygen-insensitive nitroreductases in the development of nitrofurantoin resistance in *E. coli *. We outline and categorise alterations in these three genes identified in a large collection of UK *E. coli* genomes. A proposed scoring algorithm is able to predict the level of nitrofurantoin susceptibility from genotypes, and the predictions suggest that acquired nitrofurantoin resistance is not of immediate concern in the UK. Our results also suggest a need to monitor nitrofurantoin resistance amongst *E. coli *.

## Introduction

Nitrofurantoin is a synthetic nitrofuran compound that has been widely used as a first-line antimicrobial agent for treating urinary tract infection (UTI) since 1953 and is active against a broad range of Gram-negative and Gram-positive bacteria, including *Escherichia coli *, most species of *Staphylococcus* and *Enterococcus* [1, 2]. It can reach a urinary concentration of 50–250 mg/L given a regular therapeutic or prophylactic dose while retaining negligible blood (<5 mg/L) and gastrointestinal concentrations, providing an advantage for treating UTI [3–5]. Although the antibacterial activity of nitrofurantoin is not fully understood, studies have revealed that metabolic intermediates from nitroreductase-mediated reduction of nitrofurantoin can damage DNA and RNA, as well as disrupting protein production in bacteria [6–8].

**Fig. 1.**
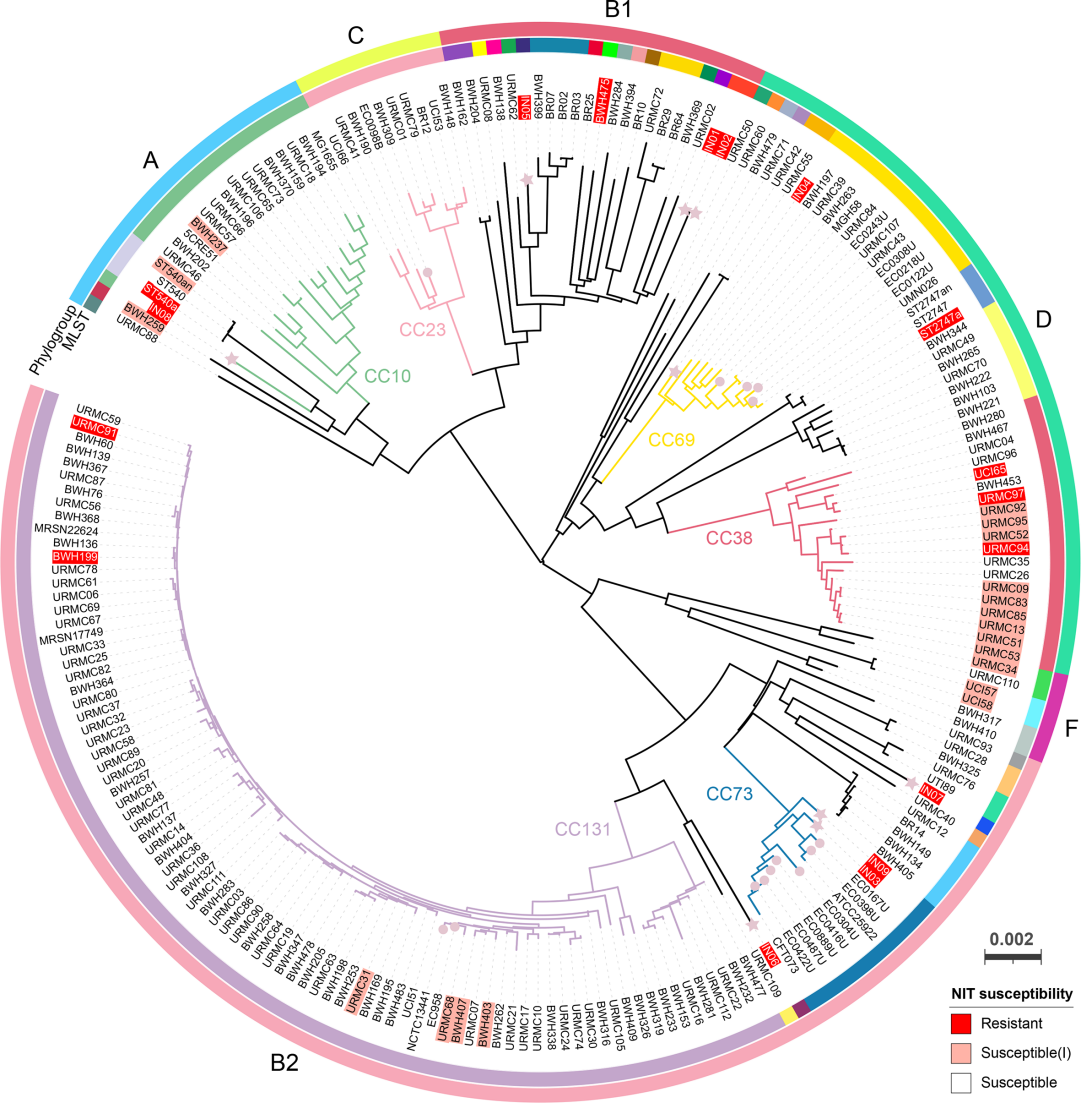
Population structure of 217 *E. coli* isolates with available nitrofurantoin (NIT) susceptibility profiles. Relatedness between these isolates is displayed by a core-genome neighbour-joining tree generated using PopPUNK. The scale bar denotes the estimated frequency of nucleotide substitutions in the core genome [42]. The tree is midpoint-rooted and drawn in a circular layout with tip labels coloured by nitrofurantoin susceptibility of isolates (Table S1). Stars highlight isolates IN01–09, and circles indicate the other 14 UK isolates. The inner track represents CCs or STs (when the CC information was unavailable) in the Achtman *E. coli* MLST scheme. CCs having ≥10 genomes are labelled over the tree. Following the EUCAST guidelines, an isolate was considered susceptible under an increased exposure to nitrofurantoin and denoted as susceptible(I) when 32 mg/L < MIC ≤ 64 mg/L (usually reported as MIC=64 mg/L). The full data can be interactively visualised on Microreact (microreact.org/project/pHwAhGZ55AL7CvXxGnziyU) [105].

*E. coli* is the predominant pathogen of uncomplicated UTI, and the increasing prevalence of isolates resistant to trimethoprim led Public Health England to recommend prescribing nitrofurantoin for UTI since November 2014 [9]. Despite clinical use of nitrofurantoin for nearly 70 years, the prevalence of nitrofurantoin resistance in *E. coli* remains relatively low in Europe. Up to 2016, fewer than 6% of *E. coli* isolates collected from urine specimens in Western, Northern, and Southern European countries were resistant to nitrofurantoin [10–14]. Separate UK-based studies showed that nitrofurantoin-resistant *E. coli* accounted for 5% of urinary and bloodstream *E. coli* isolates collected in London, during 2005–2006 and 2011–2015, despite higher prevalence of resistance (>20%) to other commonly prescribed oral antimicrobials in the same isolates [15–17].

Since *E. coli* was not widely exposed to nitrofurantoin in the UK until the adoption of new prescribing guidelines, it is worthwhile monitoring the prevalence of nitrofurantoin resistance among UK *E. coli* isolates. The reported low prevalence of nitrofurantoin resistance might be explained by the broad intracellular target range of toxic nitrofurantoin metabolites and a reported fitness cost among nitrofurantoin-resistant mutants [6, 18]. Two kinds of genetic determinants of nitrofurantoin resistance have been identified in *Enterobacteriaceae* so far: (a) loss-of-function mutations in chromosomal genes *nfsA*, *nfsB* (encoding oxygen-insensitive nitroreductases NfsA and NfsB, respectively), and *ribE* (encoding 6,7-dimethyl-8-ribityllumazine synthase, involved in the reduction of nitrofurantoin) [7, 8]; and (b) acquired gene complex *oqxAB* (encoding a multidrug efflux pump OqxAB) [19]. By contrast, bacteria with impaired DNA-repair ability showed increased susceptibility to nitrofurantoin [20, 21].

Understanding the mechanisms underpinning antimicrobial resistance (AMR) is central to predicting the impact of antimicrobial prescribing guidelines. Nevertheless, there is little or no understanding of the basis of nitrofurantoin resistance in UK *E. coli *. This is hindered by the fact that nitrofurantoin susceptibility testing is mostly limited to urinary tract isolates rather than enteric or invasive bloodstream isolates, while a vast array of whole-genome sequencing (WGS) has focussed on bloodstream isolates or isolates with multidrug-resistance phenotypes. We set out to uncover the genetic basis of nitrofurantoin resistance in nine *E. coli* isolated from UTI patients in north west London. We then screened a large genomic dataset of 12412 UK *E. coli* isolates for resistance-associated genetic alterations to enhance our understanding of the wider distribution of nitrofurantoin resistance and genetic mechanisms that influence nitrofurantoin’s interaction with *E. coli *.

## Methods

### *E. coli* isolates and nitrofurantoin susceptibility testing

Following the protocol and breakpoint from the European Committee on Antimicrobial Susceptibility Testing (EUCAST), automated disc diffusion tests for nitrofurantoin susceptibility (Oxoid CT0034B 100-µg nitrofurantoin discs, ThermoFisher Scientific, USA) in clinical urinary *E. coli* isolates were routinely performed by a microbiology laboratory of the Imperial College Healthcare NHS Trust in north west London. The laboratory serves a population of 2.5 million. In the period 2018–2019, 18 viable nitrofurantoin-resistant *E. coli* isolates were collected, however, the susceptibility result could be reproduced for only nine of these isolates (IN01–09) when frozen stock was retrieved and then retested using the same disc diffusion method. Aerobic MIC of nitrofurantoin was measured for each of the nine resistant isolates using MIC Test Strips (Liofilchem, Italy). Nitrofurantoin-susceptible *E. coli* isolate EC0098B (nitrofurantoin MIC: 32 mg/L) was chosen as a negative control. MICs were reported as per conventional two-fold series of concentration increments (2, 4, 8, … mg/L) and interpreted in accordance with the EUCAST breakpoint (v10.0) [22].

### Whole-genome DNA extraction and sequencing

Genomic DNA of the nine resistant isolates IN01–09 was extracted using a phenol/chloroform method [23], checked for integrity using gel electrophoresis, and stored at −20 °C. DNA libraries were prepared in a paired-end layout with NEBNext Ultra II FS DNA Library Prep Kit (New England BioLabs, UK) and sequenced under a 150-bp read length using Illumina HiSeq 4000 systems and HiSeq SBS Kit v4 reagents (Illumina, USA). Read demultiplexing and adapter trimming were performed by the Imperial BRC Genomics Facility using their production pipeline.

### Public *E. coli* genomes used for comparative analysis

In order to identify genetic variants related to nitrofurantoin resistance using a comparative approach, the NCBI Pathogen Detection portal (www.ncbi.nlm.nih.gov/pathogens/), NCTC 3000 Project, EnteroBase, NIHR Health Protection Research Unit (HPRU) isolate collection (BioProject accession: PRJEB20357; manuscript in preparation), and literature were searched for *E. coli* genomes from isolates with known nitrofurantoin-susceptibility profiles [8, 24–27]. Complete and draft genome assemblies were downloaded from the NCBI nucleotide database and sequence reads of unassembled genomes were downloaded from the NCBI Sequence Read Archive (SRA). In total, 208 *E. coli* genomes were obtained from the search, consisting of 196 genomes downloaded from the GenBank database (www.ncbi.nlm.nih.gov/genbank/) (Table S1 ‘NCBI’) and 12 genomes from clinical isolates collected from north west London by the HPRU in 2015–2016 (Table S1 ‘HPRU’).

### Quality control of sequence reads

Quality summaries of all read files were generated using FastQC v0.11.9 and compiled using MultiQC v1.8 [28, 29]. With Trimmomatic v0.39, the reads of IN01–09 were trimmed for an average base quality of Phred Q20 in a 5-bp sliding window and were then filtered for a minimum length of 50 bp [30]. Seqkit v0.12.0 was used for randomly sampling the filtered reads of each genome to an 80-fold coverage, assuming an average genome size of 5 Mbp [31, 32]. For reads downloaded from the SRA, case-by-case read trimming and filtering was conducted using Trimmomatic in order to deal with large variation in read quality and to obtain a coverage of less than 100 folds. Genomes having less than 40-fold coverage were excluded from further analysis. DNA contamination in reads was evaluated for each genome using Kraken v2.0.8-beta and its full bacterial database (accessed in February 2020) [33].

### *De novo* genome assembly and annotation

Reads of each genome were assembled with Unicycler v0.4.9b, which used SPAdes v3.13 for initiating assembly graphs [34, 35]. Quality of assemblies were evaluated based on summary statistics calculated by QUAST v5.0.2 [36]. Complete plasmid sequences were identified in Bandage visualisation of assembly graphs [37]. Assemblies were annotated through Prokka v1.13 with a reference protein database representing proteins extracted from all *E. coli* genomes that were publicly available in the NCBI RefSeq database by March 2020 [38]. Redundant protein sequences were removed from this database using CD-HIT v4.8.1 [39] under a minimum amino acid identity of 70%.

### Genomic comparisons of *E. coli* with known nitrofurantoin-susceptibility profiles

Multi-locus sequence typing (MLST) of the nine genomes IN01–09 was conducted using ARIBA v2.14.4 [40], and the MLST for other 208 collected genomes of *E. coli* isolates with known nitrofurantoin-susceptibility profiles was conducted using mlst (github.com/tseemann/mlst). The Achtman scheme for *E. coli *, which was downloaded from PubMLST database (pubmlst.org) with ARIBA in March 2020, was used for the MLST. To depict the overall genomic relatedness between the 217 genomes and to facilitate the selection of a reference genome for each of genomes IN01–09, pairwise core-genome and accessory-genome distances were estimated from genome assemblies with PopPUNK v2.0.2 given k-mer lengths increased from 15 bp to 23 bp by a 2-bp step size [41]. Phylogroups of the 217 genomes were determined from genome assemblies using ClermonTyping with contigs shorter than 1 kbp excluded [42]. Single-nucleotide polymorphisms (SNPs) between seemly identical genomes IN01 and IN02 were identified using Bowtie2 v2.4.1 and BCFtools v1.9 as implemented in the cgSNPs mapping pipeline (github.com/wanyuac/cgSNPs) [43–45] (Supplementary Method S1). The PopPUNK phylogenetic tree and population data were visualised using iTOL v6.3 [46].

### Identifying genetic alterations of *nfsA*, *nfsB*, and *ribE*

For each of sample genomes IN01–09, the most closely related complete genome of a nitrofurantoin-susceptible isolate was chosen from the collection of 208 genomes as a reference according to core-genome distances calculated by PopPUNK. Reads of each sample genome were then mapped to the chromosome sequence of the selected reference genome using Bowtie2 under the mode *sensitive-local*. SNPs and indels were identified from mapped reads using BCFtools when filtering out low-confidence variant calls (QUAL ≤20, DP ≤10, or MQ ≤20) as well as low-quality base calls (Phred Q <20). Moreover, SNPs and indels within repetitive regions (nucleotide identity ≥90%) identified in the selected reference sequence with nucmer v3.1 were excluded for accuracy [47], and the remaining variants in the sample genome were annotated using SnpEff v4.3t and a customised database built from the selected reference sequence [48].

Bandage v0.8.1 [37] was used to search for loci of *nfsA*, *nfsB*, and *ribE* in genome assemblies of IN01–09 and to extract the identified allele sequences. SNPs and indels were identified in these alleles using web-based megaBLAST (blast.ncbi.nlm.nih.gov) and compared to those identified through read mapping. Interruptive insertion sequences were inferred from assembly graphs using Bandage and were searched against the ISFinder database for reference insertion sequences and classification [49]. Insertion sites of these insertion sequences were determined with ISMapper v2.0.1 [50]. Copy numbers of *nfsA*, *nfsB*, and *ribE* in each sample genome were predicted from mapped reads using CNOGpro v1.1 (100-bp sliding windows and 1000 bootstrap samples per gene) [51, 52]. Translated sequences of these three genes were extracted from Prokka annotations and were aligned using the ClustalW algorithm implemented in MEGA X [53, 54]. The functional effect of each amino acid substitution was predicted using PROVEAN Protein (accessed in June 2020).

### Validating interrupted *nfsA* and *nfsB*

Insertion sequences that might have interrupted genes *nfsA* and *nfsB* in genomes IN01–03 were identified by visualising Unicycler assembly graphs using Bandage. The sequence of each interrupted region was extracted from the assembly graph using Bandage and was then confirmed using Sanger sequencing of PCR products. PCR primers (Table 1) were designed with Primer3web v4.1.0 (primer3.ut.ee) [55] to ensure a complete coverage of each template genomic region. PCR was carried out using a T100 Thermal Cycler (Bio-Rad, USA) and GoTaqDNA Polymerase (Promega, UK). PCR primers and products were sent to Genewiz (UK) for Sanger sequencing. Reads were trimmed to remove ambiguous bases before alignment.

**Table 1.** PCR primers for validating interrupted genetic regions in genomes IN01–03

Genome	Primer	Sequence	T_m_ (°C)	T_a_ (°C)	Product size (bp)
IN01, 02	L1	GTGGTGGTTATTCTTCAGGTGG	64.3	59.3	974
	R1	GAAGGGAAAGCTGCACGTAATC	65.8		
	L2	CAGCTCCACCGATTTTGAGAAC	66.9	61.7	814
	R2	CAATTTTCACCCTGCACCTCTC	66.7		
IN03	L1	CGTCCTGACTCAACCGTAAATC	64.5	58.7	895
	R1	CCTAAAATCTACTCAGCGTCGG	63.7		
	L2	GCTTGTCCCAACCTTGTTTCTG	66.7	61.5	895
	R2	CCAACCAAAGCGAGAACAAAAC	66.5		
	L3	GAAGCTCGCAATACCATAAGCC	65.6	60.6	954
	R3	CATCGAGGTGGTGTGATCAATC	66.6		

T_m_: melting temperature; T_a_: annealing temperature. L and R in primer names refer to paired left and right primers, respectively.

### Detecting acquired antimicrobial resistance genes

An ARIBA-compatible reference database of acquired AMR genes was created from the ResFinder database (commit hash: *7e1135b*) through quality filtering and sequence clustering (nucleotide identity ≥80%) using ARIBA and CD-HIT-EST v4.8.1 [39, 56]. Acquired AMR genes in genomes IN01–09 were detected from sequence reads using ARIBA and this reference database, whereas these genes in other *E. coli* genomes were identified from genome assemblies using ABRicate (github.com/tseemann/abricate) [57] and the ResFinder database without clustering.

### Searching for nitrofurantoin-resistance determinants in the UK *E. coli* population

A non-redundant reference database NITREc (github.com/wanyuac/NITREc) was created from allelic and translated sequences of *nfsA*, *nfsB*, and *ribE* in all the 217 *E. coli* isolates of known nitrofurantoin susceptibility. In particular, the allelic and protein sequences from 169 NCBI isolates and 12 HPRU isolates that had nitrofurantoin MICs ≤32 mg/L or were considered nitrofurantoin-susceptible based on disc diffusion methods were used as reference sequences for identifying wildtype alleles or proteins. CD-HIT-EST and CD-HIT were used to deduplicate reference sequences in the database. Computer scripts developed for gene detection and mutation identification are available in the NITREc code repository (github.com/wanyuac/NITREc/tree/master/Script).

Genome assemblies of UK *E. coli* isolates without available nitrofurantoin susceptibility data were then downloaded from NCBI nucleotide databases (as of August 2020; Table S2 ‘UK_public_total’) or retrieved from an ongoing NIHR HPRU study of *E. coli* bacteraemia and intestinal colonisation. Collection dates and locations of isolates were retrieved from the NCBI BioSample database, NCTC Bacteria and Mycoplasmas Browse (www.phe-culturecollections.org.uk), EnteroBase, and related literature. This information was manually inspected for accuracy and non-redundancy.

Nucleotide BLAST (megaBLAST) v2.9.0 [58] was used to identify and extract allele sequences of *nfsA*, *nfsB*, *ribE*, *oqxA*, and *oqxB* (script *searchGenes.pbs*), and the extracted sequences were then translated into protein sequences using script *translateDNA.py*. Particularly, alleles of *nfsA*, *nfsB*, and *ribE* from nitrofurantoin susceptible *E. coli* strain ATCC25922 and alleles of *oqxA* and *oqxB* from the ResFinder database were used as queries for the gene search. Since missense mutations in the start or stop codon may reduce the length of a BLAST alignment, hits showing partial or complete truncation of either codon were manually verified. CD-HIT-EST and CD-HIT were used to determine identical alleles and translated sequences, respectively. Sequence alignments were generated using Clustal Omega (www.ebi.ac.uk/Tools/msa/clustalo). Nonsense mutations were determined through comparing lengths of predicted protein sequences to the reference sequences. Missense mutations were identified in the alignments of protein sequences using script *missenseFinder.py*, which identifies amino acid substitutions in each query protein sequence by comparing it to its most closely related reference protein sequence in the NITREc database. Finally, using script *findKnownMutations.py*, missense mutations were searched for those known to be associated with nitrofurantoin resistance (Tables S3–S5) and those carried by isolates IN01–09.

**Table 2. T2:** Nonsynonymous mutations and gene interruptions in *nfsA* and *nfsB* in isolates IN01–09

Isolate	MIC (mg/L)	ST	Phylogroup	Reference	Gene	Nucleotide change	Protein change	PROVEAN score
IN01	256	1463	B1	NZ_CP035320.1 (BR02)	*nfsA*	693InsIS*1R*	Interruption	na
					*nfsB*	575G>A	G192D	−6.954 *
IN02	256	1463	B1	NZ_CP035320.1 (BR02)	*nfsA*	693InsIS*1R*	Interruption	na
					*nfsB*	575G>A	G192D	−6.954 *
IN03	128	73	B2	NZ_CP009072.1 (ATCC25922)	*nfsA*	634T>C	W212R	−12.647 *
					*nfsB*	327InsIS*10R*-like	Interruption	na
IN04	>512	69	D	NC_011751.1 (UMN026)	*nfsA*	302T>G	L101R	−3.030 *
					*nfsB*	476 : 479delTGGA	L159fs	na
IN05	256	162	B1	NZ_CP035320.1 (BR02)	*nfsA*	666delA	E223fs	na
					*nfsB*	82G>T	E28Stop	na
IN06	256	4219	B2	NZ_HG941718.1 (EC958)	*nfsA*	635G>A	W212Stop	na
					*nfsB*	281G>A	W94Stop	na
IN07	>512	919	B2	NC_007946.1 (UTI89)	*nfsA*	635G>A	W212Stop	na
					*nfsB*	137G>A	W46Stop	na
					*ribE*	151A>G	I51V	0.363
IN08	>512	685	A	NZ_CP007265.1 (ST540)	*nfsA*	121G>A	G41S	3.174
						123delT	L43fs	na
						343T>G	S115A	2.175
						350C>T	T117I	0.634
					*nfsB*	381G>A	M127I	−0.888
						458G>A	G153D	−6.995 *
IN09	256	73	B2	NZ_CP009072.1 (ATCC25922)	*nfsA*	199C>T	Q67Stop	na
					*nfsB*	113C>T	P38L	−8.693 *

MICs were determined using Etests [106]. Isolate names of reference genomes are noted by parentheses below the RefSeq accessions. Asterisks following PROVEAN scores denote deleterious mutations determined when scores ≤−2.5.

del, deletion; fs, frameshift; ins, insertion; NA, not applicable; ST, (multi-locus) sequence type.

### Evolutionary analysis of missense mutations in *nfsA*, *nfsB*, and *ribE*

For each gene, full-length, indel-free alleles were translated into protein sequences using script *translateDNA.py* (codon table: 11). Premature proteins (due to nonsense mutations) and their corresponding alleles were identified and then excluded using script *rmProteinsByLength.py*. A multi-sequence alignment of remaining alleles was generated using programme MUSCLE in software package MEGA X [59] and was then converted into a codon alignment using script *pal2nal.pl* [60]. The nucleotide nonsynonymous-substitution rate (dN) over synonymous-substitution rate (dS), denoted as *ω=dN/dS*, was used to reflect the direction and pressure of natural selection on each of the genes *nfsA, nfsB, and ribE* [61]. A maximum-likelihood (ML) estimate of ratio *ω* for each gene was then calculated from the codon alignment using a reference-free approach implemented in GenomegaMap v1.0.1 [62], assuming a constant *ω* per gene.

For each of genes *nfsA*, *nfsB*, and *ribE*, SNP sites were identified in a deduplicated set of allele sequences that were used for estimating the ω ratios — namely, untruncated alleles that only carried missense mutations. An ML phylogenetic tree was reconstructed for each gene from the alignment of deduplicated alleles. Specifically, nucleotide sequences were deduplicated using CD-HIT-EST before tree reconstruction. An extended selection of substitution models by IQ-Tree v1.6.12 (parameters: *-t BIONJ -m MF -mtree*) was performed for each gene [63]. Since the best-fit model differed between genes, candidate models of each gene were sorted in an ascending order of models’ Bayesian information criterion (BIC) scores, and the model consistently ranked <10 across all three genes was chosen for the tree reconstruction. Then an ML tree was reconstructed for each gene by IQ-Tree with the chosen model (*TIM3e+R2*), a BIONJ starting tree, ten independent runs (for selecting the tree of the greatest maximum likelihood), and 500 bootstrap replicates (for supporting branches in the selected tree).

Identification of variant sites in the sequence alignment used for reconstructing each gene tree was performed with snp-sites v2.5.1 [64]. Homoplasy sites in the alignment were identified and plotted using R package homoplasyFinder [65], which took as input the gene tree in addition to the allele alignment. A pairwise homoplasy index (PHI) was calculated from the allele alignment of each gene, and the significance of each PHI was tested for using PHIPack [66] with a 80-bp sliding window, given the null hypothesis of no recombination. Recombination within a gene was considered significant if the p-value of a PHI did not exceed 0.05.

### Predicting nitrofurantoin susceptibility from detected genetic alterations

For each *E. coli* genome that had no nitrofurantoin susceptibility data available, the prediction of nitrofurantoin susceptibility was conducted using a scoring algorithm based on sequentially categorising variations at sequence, genetic, and genomic levels. The algorithm also considers whether a gene of interest is intrinsic or acquired, and assumes that the functional loss of an intrinsic gene and the gain of a functional *oqxAB* complex are both associated with reduced nitrofurantoin susceptibility. Calculation of gene-level and genome-level scores have been implemented in NITREc helper script *scoreHitsNITR.R*, whereas manual inspections are required to determine sequence-level scores.

Firstly, at the sequence level, supposing *n_i_* BLAST hits (matched regions between query and subject sequences) of gene *i* (*i=nfsA, nfsB, ribE, oqxA, or oqxB*) are identified in a genome, a score *s_ij_* (*j=1, …, n_i_*) is assigned to each hit to represent the probability of this sequence to confer reduced nitrofurantoin susceptibility. Then an arbitrary value is assigned to the score for each hit of intrinsic genes (*nfsA*, *nfsB*, and *ribE*): *s_ij_=1*, if the sequence is truncated or interrupted (which cannot be distinguished from each other by BLAST without assembly graphs) or has a start-codon loss, a frameshift or nonsense mutation, or any missense mutation known to be associated with nitrofurantoin resistance; *s_ij_=0.1*, if the sequence only has missense mutations whose impact on nitrofurantoin susceptibility remains unknown; and *s_ij_* =0, if the sequence is identical to any known ‘wildtype’ allele present in a nitrofurantoin-susceptible isolate. On the other hand, for each hit of acquired genes (*oqxA* and *oqxB*), *s_ij_=1*, if the sequence is identical to a reference allele in the ResFinder database; *s_ij_=0.1*, if the sequence only carries missense mutations of unknown impacts on the protein function; and *s_ij_=0*, if the sequence is truncated or interrupted (No other kind of *oqxA* or *oqxB* variants were found in this study).

Secondly, a gene score *g_i_* (*i=nfsA, nfsB, ribE, oqxA, oqxB*) is determined from sequence scores. Specifically, for each intrinsic gene, *g_i_=min{s_ij_}*, where *j=1, …, n_i_*. Namely, gene *i* is believed to not confer reduced nitrofurantoin susceptibility when there is at least one wildtype allele. For acquired genes *oqxA* and *oqxB*, however, *g_i_=max{s_ij_}* (*j=1, …, n_i_*) as wildtype alleles are believed to confer reduced nitrofurantoin susceptibility. Moreover, *g_i_=1* and *g_i’_=0* when intrinsic gene *i* and acquired gene *i*’ are not detected, respectively.

Finally, a risk score *r* is calculated from gene scores for each genome. Let *g_1_*, …, *g_5_* represent gene scores of *nfsA*, *nfsB*, *ribE*, *oqxA*, and *oqxB*, respectively, then each gene score takes a value of 0, 0.1, or 1. Since the multidrug efflux pump OqxAB requires both functional components OqxA and OqxB to reduce the intracellular concentration of nitrofurantoin, the risk score is calculated by formula:

$$r=\sum_{i=1}^{3}g_{i}+min(g_{4},g_{5})$$

where $\min\left(g_{4},g_{5}\right)=1$ when $g_{4}=g_{5}=1$ , indicating a functional *oqxAB* complex. Hence *r* takes values between zero (nitrofurantoin susceptible) and four (nitrofurantoin resistant). In our study, genomes were sorted by risk scores to identify possible resistant and susceptible isolates. Predicted nitrofurantoin susceptibility was labelled and interpreted in accordance with the EUCAST terminology: S (susceptible, standard dosing regimen), if *r=0*; S(I) (susceptible, increased exposure), if *r=1*; R (resistant), if *r≥2*; S/S(I), if *r=0.1*; and S/S(I)/R, if *0.1<r<1* or *1<r<2*.

### Validating predictions of nitrofurantoin resistance

Since nitrofurantoin-susceptible HPRU isolates (based on clinical records) had been used by the prediction algorithm as references, we experimentally validated nitrofurantoin-susceptibility predictions using the remaining HPRU isolates, which had no available nitrofurantoin-susceptibility data. First, 20 HPRU isolates predicted to have reduced nitrofurantoin susceptibility were tested using Etests (Liofilchem MIC Test Strips). Second, as an exploratory step, seven additional HPRU isolates were included for Etests because these isolates carried novel mutations that had also been detected in *nfsA* or *nfsB* of IN08, or carried an alternative stop codon or novel mutation in *ribE*, where the predicted susceptibility was uncertain due to a lack of information from literature. Furthermore, disc diffusion tests (Oxoid CT0034B 100-μg nitrofurantoin discs, used as per EUCAST manual v8.0 and manufacturer’s instructions) [67] were conducted for confirming heterogeneous responses to nitrofurantoin, and MALDI-TOF mass spectrometry was used for detecting non-* E. coli* contamination. *E. coli* strain ATCC25922 was used as the quality control for Etests.

## Results

### Genomic context of clinical nitrofurantoin-resistant *E. coli* isolates from north west London

According to the EUCAST breakpoint, nitrofurantoin resistance (MIC >64 mg/L) was confirmed in nine out of 18 *E. coli* UTI isolates that had been reported as nitrofurantoin-resistant by the NHS microbiology laboratory (Table S1). Each isolate was obtained from a unique patient. Nitrofurantoin MICs of these nine isolates (IN01–09) were ≥128 mg/L[Table T2].

To contextualise the nine isolates IN01–09, genomes of a further 208 *E. coli* isolates with known nitrofurantoin-susceptibility profiles were incorporated for genomic comparisons, creating a genome collection of global nitrofurantoin-resistant and -susceptible *E. coli* isolates (Table S1). Of these 208 isolates, eight were nitrofurantoin-resistant, none of which came from the UK, and 200 were nitrofurantoin-susceptible, 14 of which came from the UK.

Population structure of the 217 isolates (including IN01–09) was interpreted using phylogroups, multi-locus sequence types (STs), and genomic similarities estimated using PopPUNK. In total, six *E. coli* phylogroups (A, B1, B2, C, D, F) and 57 STs constituting 19 clonal complexes (CCs, defined by the MLST scheme) were identified (Fig. 1, Table S1). No novel MLST allele or ST was detected. Isolates showing nitrofurantoin MICs ≥64 mg/L were seen in five phylogroups (A, B1, B2, D, and F). Isolates IN01–09 were classified into four phylogroups (A, B1, B2, D) and seven STs (Fig. 1, Table 2). Notably, SNPs and indels were not identified between the genomes of isolates IN01 and IN02, and these two genomes only slightly differed in accessory genomes by a PopPUNK accessory-genome distance (Jaccard distance based on k-mer matches) of 2.4×10^−4^, although the isolates were collected from two distinct patients at different locations, with UTI episodes that were three weeks apart. Interestingly, while the majority of nitrofurantoin-resistant isolates were sporadically distributed across these phylogroups and CCs (including major clinical lineages such as CC69, CC73, and CC131), clustering of reduced nitrofurantoin susceptibility was mainly seen among the 19 USA isolates of CC38 (Fig. 1, Table S1).

### Genetic basis of nitrofurantoin resistance in UK *E. coli* isolates IN01–09

Neither *oqxA* nor *oqxB* was identified in any of the isolates IN01–09, indicating that horizontally acquired nitrofurantoin resistance conferred by the *oqxAB* complex had not occurred in these isolates. We therefore focused on alterations in intrinsic genes *nfsA*, *nfsB*, and *ribE*. For each genome of isolates IN01–09, the most closely related complete genome (based on the PopPUNK core-genome distances) of a nitrofurantoin-susceptible *E. coli* isolate was chosen as a reference for the identification of genetic alterations.

#### Genetic alterations of *nfsA* and *nfsB*

Analysis of *nfsA* and *nfsB* confirmed the presence of a single copy of both genes in each of the nine genomes IN01–09 (Table S6 ‘Copy_number’). Further, genetic alterations affecting protein sequences were identified in both *nfsA* and *nfsB*. Altogether, these alterations consisted of missense and nonsense mutations, deletions, and interruptions by insertion sequences (Table 2). Correspondingly, predicted sequences of proteins NfsA and NfsB showed amino acid substitutions or truncations or both. Synonymous mutations in *nfsA* or *nfsB* were also detected in five genomes (Table S6). One deleterious missense mutation was predicted by PROVEAN [68] for each NfsA or NfsB sequence that only carried missense mutations (Table 2). However, these deleterious missense mutations were not present in any NfsA or NfsB sequence from the publicly available 208 *E. coli* isolates with known nitrofurantoin-susceptibility profiles. Only two deleterious missense mutations (W212R in NfsA and G192D in NfsB) were previously reported as associated with nitrofurantoin resistance (Tables S3 and S4).

**Fig. 2.**
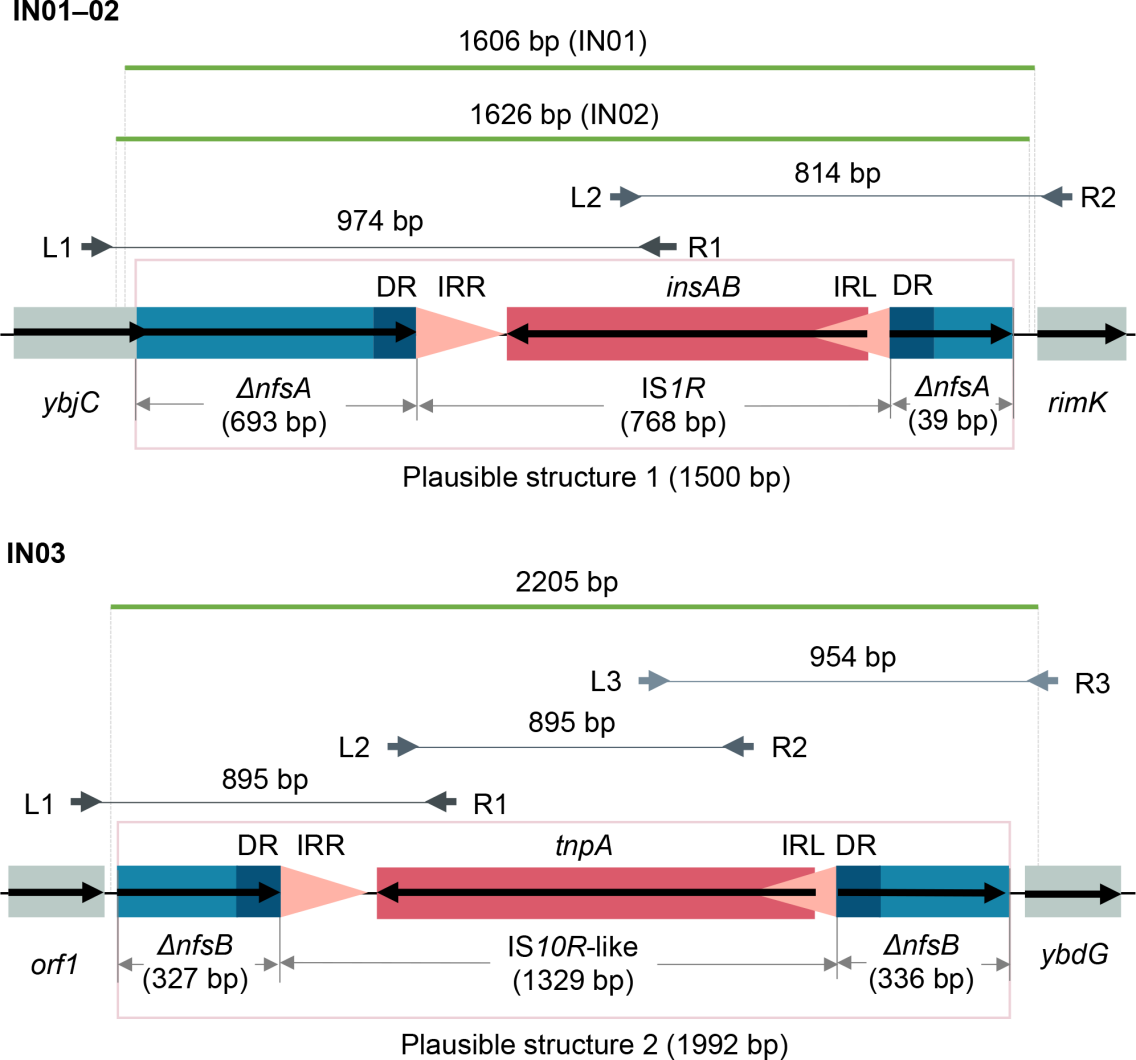
Interruption of *nfsA* and *nfsB* by insertion sequences. Orientation of each coding sequence is indicated by a black bold arrow. IRL and IRR (inverted repeat left and right) of each insertion sequence are represented by pink triangles indicating their orientations. Direct repeats (DRs) flanking each insertion sequence are denoted by dark-blue boxes. These genetic structures were confirmed by Sanger sequencing of PCR products. Paired left and right PCR primers are denoted by grey bold arrows above DNA backbones and are aligned to their source positions. Each pair of primers are connected by a grey solid line to indicate their expected PCR product. Consensus sequences of Sanger reads are denoted by green solid lines. Note that consensus sequences of IN01 and IN02 differed in lengths due to quality trimming of the reads.

Interruption of *nfsA* or *nfsB* by insertion sequences was identified in three genomes, IN01–03 (Table 2, Fig. 2). Specifically, *nfsA* in both IN01 and IN02 was interrupted by IS*1R* (768 bp, IS*1* family) encoded on the reverse complementary strand. No nucleotide divergence was seen between these two copies of IS*1R* and their reference sequence (GenBank accession: AH003427.2; region: 3877–4644). Nine-bp direct repeats (DRs) flanking IS*1R* revealed its insertion site between bases 693 and 694 of *nfsA*. Similarly, *nfsB* in IN03 was interrupted by an IS*10R*-like element (1329 bp, IS*4*-family) that differed from IS*10R* (GenBank accession: AH003348.2; bases 867–2195) by 12 nucleotides, consisting of two nucleotide substitutions in the right inverted repeat (IRR) and ten missense mutations that resulted in four amino acid substitutions in the transposase gene. This element was inserted between bases 327 and 328 of *nfsB*, as indicated by its flanking 9-bp DRs, and in an opposite orientation to *nfsB*. A search of this insertion sequence against GenBank (accessed in August 2020) found two exact matches in *E. coli* genomes of animal and clinical origins, respectively (accessions: CP009578.1 and CU928145.2). A complete Tn*10* variant (GenBank accession: AF162223.1) [69], bounded by an IS*10L*-like element and IS*10R*, was also identified in the assembly graph of IN03. Notably, this IS*10L*-like element only differed from the IS*10R*-like element of IN03 by four nucleotide substitutions in their left inverted repeats (IRLs). Assembly depths and connections of nodes consisting of the IS*10L*-like element, IS*10R*, and Tn*10* variant in the assembly graph of IN03 genome suggested high copy numbers of both insertion sequences and only a single copy of Tn*10*. Taken together, although the transposon did not integrate into *nfsB*, replicative transposition of its IS*10L* or IS*10R* component may cause the interruption of *nfsB*.

#### Genetic alterations of *ribE*

A single copy of *ribE* in each of IN01–09 genomes was predicted. Only IN07 among these nine genomes carried mutations in *ribE*, which resulted in an amino acid substitution I51V (reference genome: UTI89; Table S6 ‘Genetic_alterations’). Nonetheless, this RibE variant was identical to RibE sequences from other five nitrofurantoin-susceptible reference genomes (ATCC25922, BR02, EC958, ST540, and UMN026). Therefore, the effect of substitution I51V was considered neutral, further supported by PROVEAN prediction (score: 0.363).

### Genetic variation associated with nitrofurantoin resistance in *E. coli* from the UK

To contextualise genetic alterations identified in the nine genomes IN01–09, genomes of available 12403 UK *E. coli* isolates were retrieved, consisting of 2009 bloodstream, urine, and gut microbiota isolates from the HPRU collection (of which only 12 isolates had nitrofurantoin susceptibility information); 1509 bloodstream isolates collected by the British Society for Antimicrobial Chemotherapy (BSAC) or the Cambridge University Hospitals NHS Foundation Trust (the BSAC or CUH collection) [70]; 297 isolates from East England [71]; 162 isolates from Scotland (the SCOT collection) [72]; 85 isolates from the NCTC 3000 project [27], including historic isolates; and 8341 isolates recorded in EnteroBase [26] (Table S2).

The search for *nfsA*, *nfsB*, and *ribE* in the 12412 *E. coli* genomes (including IN01–09) with nucleotide BLAST found complete or partial matches (hits) for each gene. Of these genomes, BLAST identified 12488 hits for *nfsA* in 12410 genomes; 12393 hits for *nfsB* in 12388 genomes; and 12417 hits for *ribE* in all 12412 genomes. Notably, *nfsA* was not detected in two genomes, and *nfsB* was not detected in other 24 genomes. By contrast, 75 genomes (including IN01–02) had 2–4 hits for *nfsA* (complete or partial coding sequence); five genomes (including IN03) had two hits for *nfsB* (partial); and five genomes had two hits for *ribE* (complete). However, none of the 12412 genomes had more than one hit for any two or all of the genes *nfsA*, *nfsB*, and *ribE*.

**Fig. 3.**
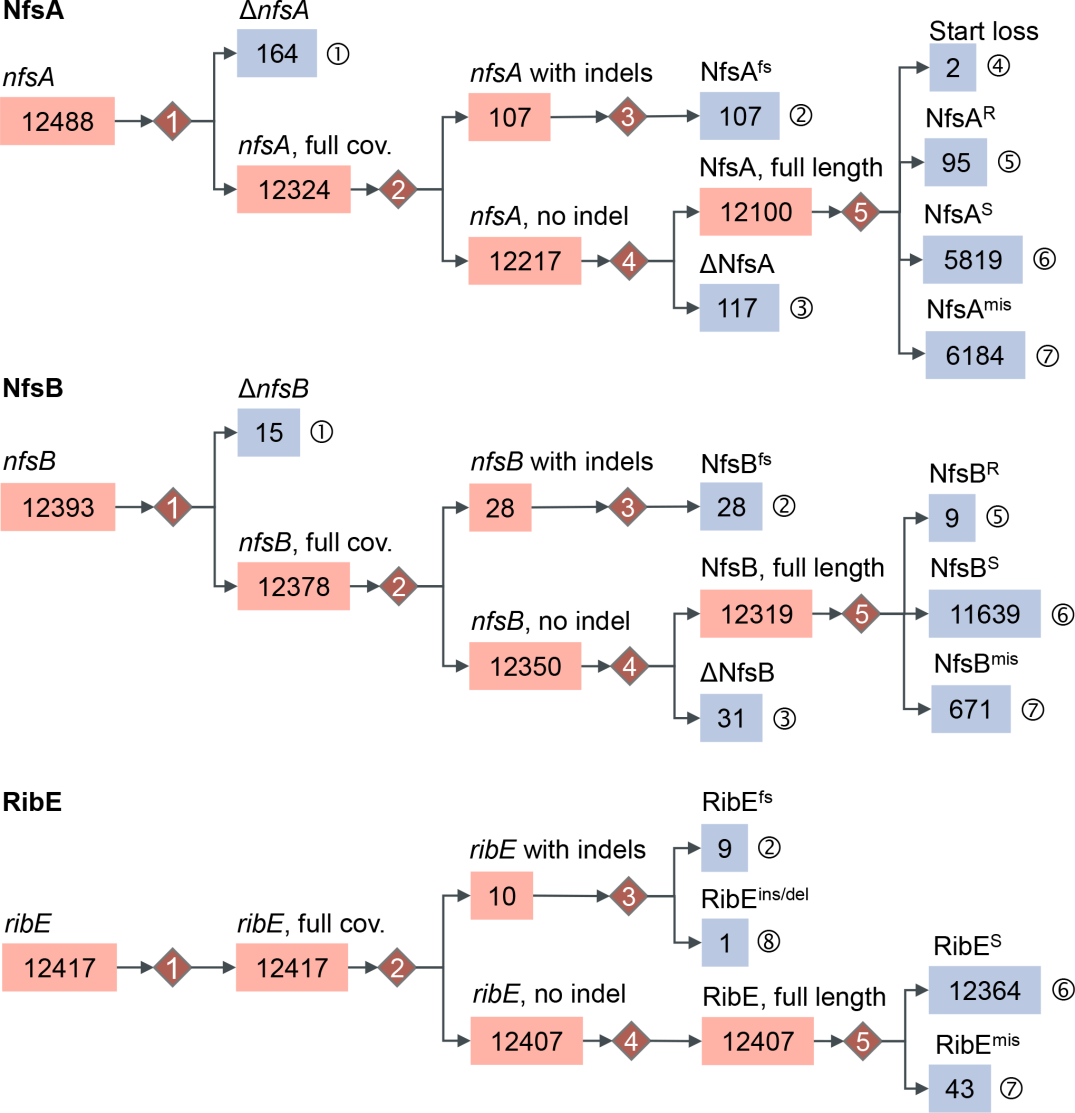
Classification of BLAST hits for *nfsA*, *nfsB*, and *ribE* from 12412 *E. coli* genomes, based on nucleotide- and protein-level comparisons. Final allele categories are labelled with ➀–➇ and shaded in blue. These categories are determined at five decision points indicated by numbered red diamonds. Numbers of alleles in each category are displayed in boxes. Categories with zero counts are omitted for convenience. Assessment at each decision point: (1) Does the allele sequence cover 100% of its reference sequence? (2) Is there any gap opened in the sequence alignment? (3) Is there a frameshift mutation or insertion/deletion of any codon? (4) Is there any nonsense mutation? (5) Does the predicted protein sequence carry no mutation, or any known resistance-associated amino acid substitution, or any other amino acid substitution? Notations: Δ, partial sequence compared to its reference; cov., query coverage; fs, frameshift mutation; ins/del, insertion or deletion of amino acid residues; R, missense mutations known to be associated with nitrofurantoin resistance; S, proteins identical to those in nitrofurantoin-susceptible isolates; mis, missense mutations of unknown phenotypical impacts.

A decision tree was then developed to categorise the hits for each of genes *nfsA*, *nfsB*, and *ribE* based on predicted protein sequences (Fig. 3). Specifically, 164 partial sequences (39–714 bp) of *nfsA* (wildtype length: 723 bp) were identified, which failed to encode complete 240-aa (amino acid) NfsA proteins. Frameshift mutations were present in all 107 alleles with indels, resulting in predicted proteins of 18–247 aa. Nonsense mutations identified in 117 alleles encoded partial NfsA proteins of 23–234 aa. Interestingly, amongst missense mutations, the start codon ATG of *nfsA* was altered in 14 genomes: mutation M1T (2T>C) was found in an allele shared by two BSAC isolates eo393 and eo2899 (collected in 2002 and 2010, respectively), causing a loss of the start codon; start-codon substitution M1I (3G>A) was found in five *nfsA* alleles from 12 genomes (Table S7) and was considered deleterious to protein structure according to the PROVEAN prediction (score: −3.149).

Genetic alterations of *nfsB* (654 bp) displayed a similar pattern to *nfsA*. Particularly, 15 partial sequences (35–647 bp) were identified and no start-codon loss was seen amongst missense mutations (Table S7). By contrast, *ribE* (471 bp) displayed lower diversity than the other two genes, with no partial allele sequence or nonsense mutation identified.

Among *ribE* alleles identified in the 12412 genomes, a stop-codon substitution (TGA >TAA) owing to missense mutation 470G>A was found in two *ribE* alleles that were present in 31 genomes. Both alleles encoded the same RibE protein as the nitrofurantoin-susceptible strain ATCC25922 (NCBI protein accession: WP_001021161.1). A novel deletion of four amino acid residues (KAGN, from position 132 to 135 in the reference RibE sequence from the ATCC25922 genome) was predicted from one (carried by isolate EC0430U) of ten *ribE* alleles that possessed indels. Notably, this deletion overlapped the deletion of amino acid residues 131–134 (TKAG, from the same reference RibE sequence) that is known to reduce nitrofurantoin susceptibility [8]. Putatively deleterious missense mutations of RibE were identified in three genomes: EC0340B (A16V), EC0444B (A34T), and EC1165B (T131S), with PROVEAN scores of -3.406,–3.893, and −2.742, respectively (Table S7).

Acquired resistance genes *oqxA* (1176 bp) and *oqxB* (3153 bp) were rare among the 12412 genomes: both genes were detected in only one genome (eo1692 from the BSAC collection), with exact matches to their reference sequences in the ResFinder database [56]. No other hit for either gene was obtained given a minimum nucleotide coverage and minimum query coverage of 70% and 80%, respectively.

At the gene level, *nfsA*, *nfsB*, and *ribE* alleles in 43% of the 12412 genomes perfectly matched to those in known nitrofurantoin-susceptible or wildtype *E. coli* isolates; 54% were one-gene mutants or single mutants (namely, each had a mutated *nfsA*, *nfsB*, or *ribE* gene); 3% were two-gene mutants or double mutants; and 0.05% were three-gene mutants or triple mutants (Tables 3 and S7). Of note, isolates NCTC11117 and NCTC11132, each carried a wildtype allele and a frameshifted allele of *nfsA*, were not considered as mutants because of the presence of the wildtype alleles (Table S7).

**Table 3.** Numbers of *E. coli* isolates with mutated *nfsA*, *nfsB*, and *ribE* genes across genome collections

Mutation	BSAC	CHU	HPRU	LTCF	NCBI	NCTC	SCOT	IN01-09	Sum
None	758	308	1390	266	2466	43	127	0	5358
One-gene	269	89	520	31	5724	32	27	0	6692
Two-gene	67	18	99	0	146	9	8	9	356
Three-gene	0	0	0	0	5	1	0	0	6
Sum	1094	415	2009	297	8341	85	162	9	12412

#### Evolutionary patterns of nucleotide substitutions in *nfsA, nfsB*, and *ribE*

Noticing the differences between the observed allelic diversities of these three genes in the 12412 *E. coli* genomes (Fig. 3), we hypothesised that *ribE* was under stronger negative or purifying selection than *nfsA* and *nfsB.* The estimated *dN/dS* ratio *ω* was 0.607812, 0.40739, and 0.179068 for *nfsA, nfsB,* and *ribE,* respectively*,* indicating that all three genes were under purifying selection with ascending strengths in the order *nfsA<nfsB<ribE*.

After obtaining unique alleles by deduplicating identical, full-length hits from the 12412 genomes, 231 (32% of 723 bp), 184 (28% of 654 bp), and 86 (18% of 471 bp) SNP sites were found in 328 *nfsA* alleles (from 12098 hits), 231 *nfsB* alleles (from 12350 hits), and 88 *ribE* alleles (from 12407 hits), respectively (Figs S1–S3). Despite this variation, phylogenetic trees of these genes generally showed extremely low bootstrap values (for instance, 0–2) in the majority of ancestral branches (Dataset S1), suggesting a lack of consensus phylogenetic information in these alleles for reliably reconstructing the evolutionary history of each gene.

PHIs calculated from the allele alignment of each gene revealed significant homoplasy in *nfsA* (*P*-value: 0.0434) and *nfsB* (*P*-value: 0.0363) but not in *ribE* (*P*-value: 0.736). Comparisons of each allele alignment to its corresponding gene tree identified 82, 50, and 12 homoplasic SNP sites in *nfsA*, *nfsB*, and *ribE*, respectively (Figs S1–S3).

### Prediction of nitrofurantoin susceptibility and experimental validation

For each of the 1997 HPRU isolates without known nitrofurantoin-susceptibility profiles, nitrofurantoin susceptibility was predicted from sequence categories (as illustrated in Fig. 3) of intrinsic genes *nfsA*, *nfsB*, *ribE* and presence-absence of both acquired genes *oqxA* and *oqxB* using the scoring algorithm. Since neither *oqxA* nor functional *oqxB* was detected in any of these isolates, the algorithm gave a risk score *r* (*0≤r≤4*) to each isolate as a summary of its three-gene (*nfsA*, *nfsB*, and *ribE*) sequence-category profiles or genotypes. We hypothesised that a higher score would predict a greater reduction in the nitrofurantoin susceptibility of an isolate, and vice versa.

Using *r≥1* as a criterion, a non-redundant set of 62 HPRU *E. coli* isolates (one isolate per patient) were predicted to have reduced nitrofurantoin susceptibility (Table S7). Since RibE sequences of these isolates were identical to those of wildtype RibE proteins in nitrofurantoin-susceptible isolates, 16 genotypes associated with reduced nitrofurantoin susceptibility were identified based on *nfsA* and *nfsB* sequences only (Table 4).

**Table 4.** The *nfsA-nfsB* genotypes of 62 non-redundant HPRU *E. coli* isolates with nitrofurantoin-resistance risk scores ≥1

***nfsA \ nfsB***	**Wildtype**	**Missense, known**	**Missense, unknown**	**Nonsense**	**Frameshift**	**Fragmented**	**Absent**
Wildtype	0	1 (1)	0	0	1 (1)	0	0
Missense, known	9 (3)	0	3 (2)	1 (1)	0	0	1 (1)
Missense, unknown	0	0	0	0	2 (1)	0	1 (1)
Nonsense	13 (1)	1 (1)	1 (1)	0	1 (1)	0	0
Frameshift	13 (1)	0	0	0	0	0	0
Fragmented	11 (1)	1 (1)	2 (2)	0	0	0	0

Each entry shows the number of isolates with each genotype, with the number of isolates selected for experimental validation displayed between parentheses. Row and column names represent genotypes of *nfsA* and *nfsB*, respectively. A gene was considered fragmented when it was interrupted or truncated. In total, 16 genotypes had at least one isolate each, from which 20 isolates were selected for the validation.

Assuming nonsense or frameshift mutations observed in this study always cause a loss of function, 20 representative isolates of the 16 genotypes were selected from the 62 isolates as a ‘prediction group’ for quantitative antimicrobial susceptibility testing (Table 5). Specifically, one representative was chosen for each genotype that did not involve any missense mutation; otherwise, one representative was chosen for each missense mutation. In this group, all 12 double mutants carried at least one resistance-associated genetic alteration (*1<r≤2*). Nitrofurantoin susceptibility was correctly predicted for all these 12 isolates. Notably, the double mutants were highly resistant to nitrofurantoin (MIC ≥256 mg/L) when both genetic alterations were known to be associated with nitrofurantoin resistance. Predictions for the eight single mutants of resistance-associated genetic alterations (*r=1*) were less reliable than those for double mutants, with five single mutants being more susceptible to nitrofurantoin than predicted (Table 5). Isolates EC394_9, EC0880B, and EC0026B showed heterogenous responses to nitrofurantoin as a few colonies of resistant *E. coli* appeared within inhibition zones. MALDI-TOF did not detect any non-* E. coli* contamination in cultures of these three isolates.

**Table 5.** Genotypes and confirmed nitrofurantoin susceptibility of 27 *E. coli* isolates in test groups

Group	Isolate	NfsA/*nfsA* (240 aa)	NfsB (217 aa)	RibE/*ribE* (156 aa)	*R*	Susceptibility		MIC (mg/L)
						Predicted	Observed	
Prediction (20 isolates)	EC0064B	G154E	Wildtype	Wildtype	1.0	S(I)	S(I)	64
	EC0890B	H11Y	Wildtype	Wildtype	1.0	S(I)	S	16
	EC1069B_1	G126R	Wildtype	Wildtype	1.0	S(I)	S	32
	EC394_9	Q67Stop	Wildtype	Wildtype	1.0	S(I)	S(I)	64 *
	EC0880B	1 : 421int413 : 723	Wildtype	Wildtype	1.0	S(I)	R	256 *
	EC1161B	I228fs	Wildtype	Wildtype	1.0	S(I)	S	32
	EC0932B	Wildtype	F84S	Wildtype	1.0	S(I)	S	4
	EC1187B	Wildtype	Y183fs	Wildtype	1.0	S(I)	S	32
	*EC0026B*	Q113Stop	H80Y	Wildtype	1.1	S(I) or R	S(I)	64 *
	*EC0067B*	1 : 308int434 : 723	W46R	Wildtype	1.1	S(I) or R	R	>512
	*EC0179B*	G126R	W94Stop	Wildtype	2.0	R	R	>512
	*EC0328B*	G126R	Absent	Wildtype	2.0	R	R	256
	*EC0363B*	R133C	T88fs	Wildtype	1.1	S(I) or R	R	128
	*EC0439B*	H11Y	W46R	Wildtype	1.1	S(I) or R	R	>512
	*EC0553Bo*	S38F	Absent	Wildtype	1.1	S(I) or R	R	>512
	*EC0629B*	1 : 52del	G192S	Wildtype	2.0	R	R	512
	*EC0644B*	1 : 69del	P209L	Wildtype	1.1	S(I) or R	S(I)	64
	*EC0812U*	W159Stop	L22fs	Wildtype	2.0	R	R	>512
	*EC0856B*	G131D	K205E	Wildtype	1.1	S(I) or R	R	>512
	*EC1131B*	E75Stop	**G192D**	Wildtype	2.0	R	R	>512
Exploration (7 isolates)	EC6002_8	G41S	Wildtype	Wildtype	0.1	S or S(I)	S	8
	*EC1146B*	G66R	Wildtype	Alt. stop	0.1	S or S(I)	S	32
	EC6125_8	Wildtype	**M127I**	Wildtype	0.1	S or S(I)	S	16
	EC0340B	Wildtype	Wildtype	A16V	0.1	S or S(I)	S	16
	EC0430U	Wildtype	Wildtype	132 : 135del	0.1	S or S(I)	S(I)	64
	EC0444B	Wildtype	Wildtype	A34T	0.1	S or S(I)	S	32
	EC1165B	Wildtype	Wildtype	T131S	0.1	S or S(I)	S	8
Control	ATCC25922	Wildtype	Wildtype	Wildtype	0.0	S	S	16

Lengths of wildtype NfsA, NfsB, and RibE are noted beneath protein names. Names of isolates carrying two mutations (namely, double mutants) are italicised. Known genetic alterations associated with nitrofurantoin resistance ([Table T2]missense mutations shown in Tables S3–S5, nonsense mutations, frameshift mutations, gene interruptions and truncations) are shaded in red. Genetic alterations identified in IN01–09 genomes are boldfaced. Asterisks indicate isolates of which colonies within inhibition zones were seen and considered when determining the MICs. Interpretation of MICs: S, susceptible (MIC ≤32 mg/L); S(I), susceptible, under increased exposure to nitrofurantoin (32 mg/L < MIC ≤ 64 mg/L); R, resistant (MIC >64 mg/L). The control strain was not used for evaluating the prediction accuracy because its *nfsA*, *nfsB*, and *ribE* alleles were used as references by the prediction algorithm.

alt. stop, alternative stop codon; del, deletion or truncation, with its start and end nucleotide positions noted on the left side; int, gene interruption, with nucleotide positions (start : end) of remnants related to their wildtype references noted aside.

Seven additional HPRU *E. coli* isolates were included as an exploratory group to examine potential effects of novel mutations on nitrofurantoin susceptibility (Table 5): NfsA or NfsB of two isolates had a missense mutation that had also been identified in isolate IN08; *ribE* of one isolate was terminated by an alternative stop codon TAA; and other four isolates had mutations in RibE, while genomes of all these four isolates encoded wildtype NfsA and NfsB. A nitrofurantoin-resistance risk score of 0.1 was assigned to each isolate by the prediction algorithm to denote presence of a novel mutation. Upon testing, all seven isolates were susceptible to nitrofurantoin (MICs ≤64 mg/L), matching our predictions. None of the six missense mutations resulted in nitrofurantoin resistance. By contrast, isolate EC0430U, which had a deletion of KAGN^132–135^ in RibE, showed an MIC of 64 mg/L, exceeding the nitrofurantoin MIC of a previously reported isolate having an overlapping 4-aa deletion in RibE [8].

### Predicted occurrences of reduced nitrofurantoin susceptibility in UK *E. coli* bloodstream isolates

As data relating to nitrofurantoin susceptibility amongst bacteriaemia *E. coli* isolates is seldom available, possible occurrences of reduced nitrofurantoin susceptibility were calculated for a non-redundant collection of 2253 bacteriaemia *E. coli* isolates that were collected in 2001–2016 from across the UK. This collection comprised 582 HPRU isolates (one isolate was selected per ST per patient), 1509 BSAC or CUH isolates, and 162 SCOT isolates. Overall, 142 (6.3%) of the 2253 isolates were predicted to show reduced nitrofurantoin susceptibility. Specifically, 123 (5.5%) single-mutation isolates had a resistance-associated alteration in either *nfsA* (117 isolates, 5.2%) or *nfsB* (six isolates, 0.3%), and 19 (0.8%) double-mutation isolates had resistance-associated alterations in both *nfsA* and *nfsB*.

## Discussion

In this study, we have explored genetic determinants of nitrofurantoin resistance in UK *E. coli* starting with nine sequenced nitrofurantoin-resistant clinical isolates and a comparative genomics approach. We found four types of alterations in two intrinsic, oxygen-insensitive nitroreductase genes (*nfsA* and *nfsB*) that are known to be associated with nitrofurantoin resistance in *E. coli *: gene interruptions by insertion sequences, frameshift mutations caused by indels, nonsense mutations and missense mutations both caused by single-nucleotide substitutions. Notably, each of these nine isolates had alterations in both *nfsA* and *nfsB*, equivalent to double-step or multi-step mutants of both genes, and hence explaining high nitrofurantoin MICs (≥128 mg/L) of these isolates [73].

Both *nfsA* and *nfsB* had at most one missense mutation identified in genomes of isolates IN01–07 and IN09, and these mutations were predicted to be deleterious to protein functions by PROVEAN (Table 2). Mutation W212R of NfsA in isolate IN03 was previously identified in an Iranian nitrofurantoin-resistant *E. coli* isolate EC168 (MIC ≥512 mg/L), in which no functional *nfsB* gene was detected [74]. Since nucleotide substitutions differ between IN03 (634T>C) and EC168 (634T>A), this mutation may have arisen independently in each isolate. W^212^, located in α-helix α10, forms the active site of NfsA (Protein Data Bank or PDB accession for protein structure: 1F5V) with the hydrophobic side chain of tryptophan and is conserved when compared with the NfsA counterparts in *Vibrio harveyi* and *Bacillus subtilus* [75, 76]. A functional disruption can hence be anticipated when substituting this side chain with the positively charged side chain of arginine. The other missense mutation of NfsA identified in our study, L101R in isolate IN04, is novel and, as in the case of EC168, this mutation possibly disrupts the function of NfsA because IN04 was highly resistant to nitrofurantoin (MIC >512 mg/L) and did not possess a functional *nfsB* gene. Since L^101^ is part of α-helix α6 in the central domain of NfsA [75, 76], substitution of this hydrophobic, non-polar residue with arginine may have a disruptive impact on the protein structure and function.

Mutation G192D in NfsB from isolates IN01 and IN02 was previously identified in a nitrofurantoin-resistant *E. coli* isolate (collected in 1999–2000) having a deletion in *nfsA* and showing a nitrofurantoin MIC of 128 mg/L [73, 77]; substitution of G^192^ with alanine (G192A) has also been associated with nitrofurantoin resistance [73]. Located at the end of the fourth β-sheet of NfsB (PDB accession: 1DS7), G^192^ constitutes the hydrophobic core that accommodates co-factor flavin mononucleotide [78] and is in close spatial proximity of negatively charged residue D^160^. Therefore, substitution of the neutral, hydrophobic residue G^192^ with an aspartic acid residue possibly affects the protein formation as well as its function. A similar impact of the NfsB mutation G153D in isolate IN08 on the protein function is anticipated based on the same difference in hydrophobicity of amino acid residues.

Nonsense mutations, frameshift mutations, and insertion-sequence-mediated interruptions of *nfsA* or *nfsB* are also common loss-of-function genetic alterations causing nitrofurantoin resistance [73, 74, 79], and we found examples of all these alterations in isolates IN01–09 (Table 2). The interruption of *nfsA* by an IS*1*-family insertion sequence has been reported by three other studies, two of which also identified insertion of IS*1* in *nfsB* [7, 8], while the third study identified integration of the composite transposon Tn*10* into *nfsA* [73]. Interestingly, the position and flanking nucleotides of the IS*1* insertion site differ among all three studies, indicating variability of IS*1* in gene inactivation and being consistent with the known AT-rich target-site specificity of IS*1* [80]. In comparison, the interruption of *nfsB* by a novel IS*10R*-like insertion sequence had not been reported before, although a different IS*4*-family insertion sequence IS*186* was found in disrupted *nfsA* [7]. Since IS*10*-group elements comprise both ends of Tn*10*, the presence of Tn*10* in isolate IN03 implies transposition of IS*10R* or its IS*10L*-like element from Tn*10* to *nfsA* or *nfsB*, conferring reduced nitrofurantoin susceptibility to host bacteria. Nevertheless, we anticipate that it is less likely for IS*10L* (the left end of Tn*10*) to show the same behaviour as IS*10R* because its transposition function is much weaker than the latter [81]. We might see a growing frequency of insertion-sequence-mediated nitrofurantoin resistance in the future, if copy numbers of IS*1*- and *IS4*-family elements in *E. coli* genomes are undergoing similar accumulation trajectories as those in *Shigella* species [82].

This study has revealed variations of genes *nfsA*, *nfsB*, and *ribE* among UK *E. coli* isolates, and such variations are consistent with the 194 NCBI isolates (excluding UK isolates EC958 and NCTC13441) collected in other countries. Identified alleles of these genes (Fig. 3) can be summarised into two primary types: functional alleles and pseudogenes. The latter can result from gene truncation, insertion-sequence-mediated gene interruption, frameshift mutations, nonsense mutations, as well as deleterious missense mutations, and confers reduced nitrofurantoin susceptibility to *E. coli *.

The use of a large and diverse genome collection enabled us to discover evolutionary patterns of *nfsA*, *nfsB*, and *ribE*. Comparisons between dN/dS ratios of these genes support our hypothesis that *ribE* is subjected to the strongest pressure of negative selection whereas *nfsA* is subjected to the weakest. Not only does this variation in selective pressures explain the observed diversity differences between sequence variations of these three genes in both this study and literature (Tables S3–S5), but also explains why stepwise reduction of *E. coli* nitrofurantoin susceptibility usually starts from a mutation in *nfsA* (the first-step mutation) — a phenomenon that has been reported by several studies [7, 8, 73]. The differences in gene lengths of *nfsA* (723 bp), *nfsB* (654 bp), and *ribE* (471 bp) may also contribute to this order of stepwise mutations, as a long gene might be more likely to gain spontaneous mutations than a short gene within the same cell. Notably, *nfsA* and *nfsB* are classified as non-essential genes of *E. coli *, whereas *ribE* is considered as an essential gene by the PEC database (shigen.nig.ac.jp/ecoli/pec) [83]. The higher level of evolutionary conservation of *ribE* compared with *nfsA* and *nfsB* appears to represent a greater degree of functional constraint on its sequence [84]. Nonetheless, expression levels of these three genes might also be important to consider [85].

Homoplasic SNPs were identified in *nfsA*, *nfsB*, and *ribE* from *E. coli* genomes in our collection (Figs S1–S3), and recombination within *nfsA* and *nfsB* was also inferred by the pairwise homoplasy indexes. Both factors may contribute to the lack of congruent phylogenetic signals in alleles of each gene (shown by low bootstrap values of branches in each gene tree), indicating that, in addition to recombination, resistance mutations can arise independently in each gene across the *E. coli* population. Because the acquired resistance gene complex *oqxAB* was extremely rare in our *E. coli* genome collection, *de novo* mutations of chromosomal genes *nfsA*, *nfsB*, and *ribE*, and insertion-sequence-mediated interruptions of *nfsA* and *nfsB* may constitute the main source of reduced nitrofurantoin susceptibility in UK *E. coli* isolates.

We developed a decision-tree based algorithm for predicting nitrofurantoin susceptibility from five loci (*nfsA*, *nfsB*, *ribE*, *oxqA*, and *oqxB*) that are known to be involved in nitrofurantoin resistance. Nitrofurantoin-susceptibility testing showed that the algorithm correctly predicted susceptibility levels for double mutants with known resistance-associated genetic alterations and for single mutants with novel genetic alterations (Table 5). Consistent results were obtained when applying the algorithm to non-UK *E. coli* isolates (Tables S7 and S8). The unexpected lower MICs of isolates carrying single resistance-associated alterations (which include confirmed loss-of-function alterations, such as nonsense and frameshift mutations, gene interruptions and truncations) in *nfsA* or *nfsB* suggests that impaired function or production of both oxygen-insensitive nitroreductases NfsA and NfsB may be required to render *E. coli* resistant to nitrofurantoin, or there are unknown mechanisms rendering *E. coli* susceptible to nitrofurantoin. This discrepancy between the predictions and susceptibility profiles also implies that we might have overestimated the occurrence of reduced nitrofurantoin susceptibility in the collection of sequenced *E. coli* bloodstream isolates from the UK, although the estimate of 6.3% is close to the reported prevalence (5%) of nitrofurantoin-resistant *E. coli* in England by 2019 [15, 16, 86].

To provide reference information for future research, we have developed database NITREc (github.com/wanyuac/NITREc), which will facilitate searching for the most closely related reference sequence of a query allele of *nfsA*, *nfsB*, or *ribE*, minimising the number of reported variants and reducing noise in the results. We anticipate improvement in accuracy of both variant identification and susceptibility prediction when new references are incorporated into the database in the future.

From our analysis of a wider, global collection of 217 *E. coli* isolates with known nitrofurantoin susceptibility, nitrofurantoin-resistant isolates (MICs >64 mg/L) were sporadically distributed in phylogroups A, B1, B2, and D (Fig. 1). These phylogroups cover CCs that are common causes of *E. coli* UTI and bacteraemia (e.g. CC69, CC73, CC131) and/or have extensive AMR profiles (e.g. CC10, CC38, CC69, CC131) [87–90]. Since 6–13 % of nitrofurantoin intake reaches the colon [91], the low gastrointestinal nitrofurantoin concentration may favour *E. coli* mutants of reduced nitrofurantoin susceptibility via direct or bystander selection [8, 92], paving the way for emergence and spread of nitrofurantoin resistance regardless of the clonal background.

Notably, the core-genome identity between ST1463 isolates IN01 and IN02 points more definitively to a transmissive potential or common source for these nitrofurantoin-resistant isolates, albeit further epidemiological data were not available relating to additional patient risk factors or any common exposures. There is a low prevalence (≤6%) of gastrointestinal colonisation by nitrofurantoin-resistant *E. coli* in human and farm animals [93–95], and it is hypothesised that these carriage bacteria may colonise the urethra via periurethral contamination and progress to UTI [96]. Horizontal transmission of nitrofurantoin-resistant isolates might be feasible if the resistance does not compromise bacterial survival or pathogenicity. This potential might be the case of isolates IN01 and IN02, as ST1463 *E. coli* has adapted to a wide range of niches (including human, companion animals, livestock, poultry, and environmental matrices), carries diverse AMR and virulence determinants, and is reported to transmit between human and animals [97–102].

This study has several limitations. First, the impact of novel missense mutations in *nfsA* and *nfsB* on nitroreductase function and bacterial fitness were not experimentally confirmed, for example, through mutagenesis experiments. Such confirmation is desirable as the observed variation in MICs of single-mutation isolates carrying missense mutations indicates that PROVEAN-predicted deleterious *nfsA*/*nfsB* mutations may not cause reduced nitrofurantoin susceptibility and their effects may be offset by other susceptibility mechanisms. Second, epidemiological analysis of nitrofurantoin-resistant *E. coli* is limited owing to a lack of available nitrofurantoin-susceptibility profiles of *E. coli* isolates from the UK or abroad. As such, several key clinical questions have not been addressed in this study, including the association of STs or infection sites (for instance, bloodstream and urinary tract) with nitrofurantoin resistance, co- or cross-resistance between nitrofurantoin and other antimicrobials, and temporal trend of nitrofurantoin resistance in the UK. Third, regulatory elements (such as promoters) of the nitroreductase system and DNA-repair mechanisms were not investigated in this study. Furthermore, untargeted analysis was not carried out for identifying novel genetic mechanisms of nitrofurantoin resistance due to the small number of nitrofurantoin-resistant *E. coli* isolates that were available to this study.

## Conclusions

We predict the major cause of nitrofurantoin resistance in UK *E. coli* to comprise sporadic *de novo* mutations in chromosomal genes *nfsA*, *nfsB*, and *ribE*; and interruptions of *nfsA* and *nfsB* by insertion sequences. Accordingly, clonal expansion of resistant mutants, mobilisation of insertion sequences, and selection of resistant clones in the presence of nitrofurantoin are believed to be three driving forces in the evolution of nitrofurantoin-resistant *E. coli* in the UK. Previous reports have suggested that *nfsA* and *nfsB* mutations are associated with significant fitness cost that may obstruct propagation of resistant *E. coli* [73, 103]. However, it is notable that isolates investigated in these reports and the current research were all identified from clinically significant infections, implying a fitness to survive in enteric microbiota and to survive the host immune response in the lower urinary tract. This fitness was also supported by the possible community transmission identified with clonal isolates IN01 and IN02. The same nitrofurantoin-resistance determinants of IN01–09 were also identified in bloodstream isolates, underlining the ability of these *E. coli* to adapt to circumstance.

It is unclear whether nitrofurantoin resistance will become more prevalent among *E. coli* in the UK as a result of increased community nitrofurantoin exposure following the changes in national prescribing guidelines. As such, routine nitrofurantoin susceptibility testing and WGS of *E. coli* isolates are needed to monitor this trend. The tools provided in our study will facilitate future WGS-based surveillance. Further work is required to assess the fitness cost of resistance-associated genetic alterations and to identify novel mechanisms of nitrofurantoin resistance.

## Supplementary Data

Supplementary material 1

Supplementary material 2

Supplementary material 3

Supplementary material 4

Supplementary material 5

Supplementary material 6

Supplementary material 7
