# Supplementary material for: Alterations in chromosomal genes nfsA, nfsB, and ribE are associated with nitrofurantoin resistance in Escherichia coli from the United Kingdom
Source: Microb Genom. 2021 Dec 3;7(12):000702. doi: 10.1099/mgen.0.000702 (PMC8767348; doi:10.1099/mgen.0.000702)
Supplement: Supplementary material 1 [file mgen-7-0702-s001.pdf]

Alterations in Chromosomal Genes *nfsA*, *nfsB*, and *ribE* Are Associated with Nitrofurantoin Resistance in *Escherichia coli* from the UK

Supplementary methods for read mapping

Yu Wan, Ewurabena Mills, Rhoda C.Y. Leung, Ana Vieira, Xiangyun Zhi, Nicholas J. Croucher, Neil Woodford, Elita Jauneikaite, Matthew J. Ellington, and Shiranee Sriskandan
September 2021

Given the high similarity in contig-length distributions between *SPAdes* genome assemblies of these two isolates (Fig. S4), IN01 was chosen as the reference genome for read mapping as its total length was 4.7 kbp greater than that of IN02.

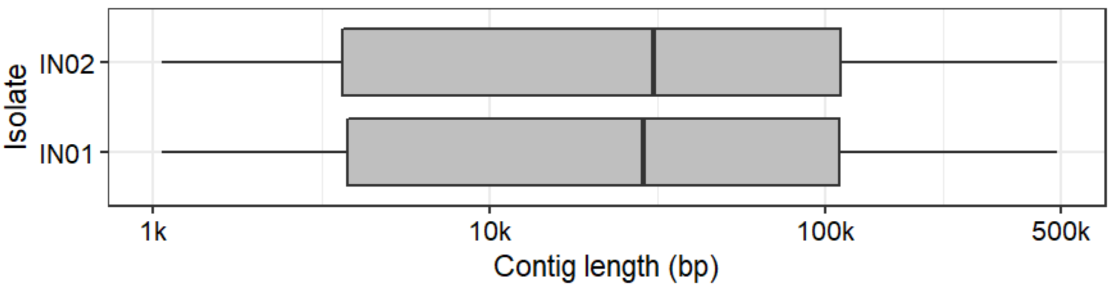

Fig. S4. Box plots showing the contig-length distributions of IN01 and IN02 genome assemblies.

To reduce mapping errors caused by repetitive regions that could not be resolved using short reads, contigs of IN01 having lengths ≤ 1 kbp and *SPAdes* assembly depths ≥ 3 folds were filtered out before read mapping (Fig. S5). The *cgSNPs* pipeline was configured to run *Bowtie2* under the mode sensitive-local, run *BCFtools mpileup* for a minimum base quality of 20 and a minimum mapping quality of 30, and to filter the result VCF file by criteria "*DP>=10 & QUAL>=30 & MQ>=30 & AF==1*".

The same configurations were also applied for detecting variants between genomes BWH453 and UCI65 (the reference genome). The variants were annotated using *SnpEff v4.3t* in an attempt to explain the unpredicted nitrofurantoin resistance of UCI65.

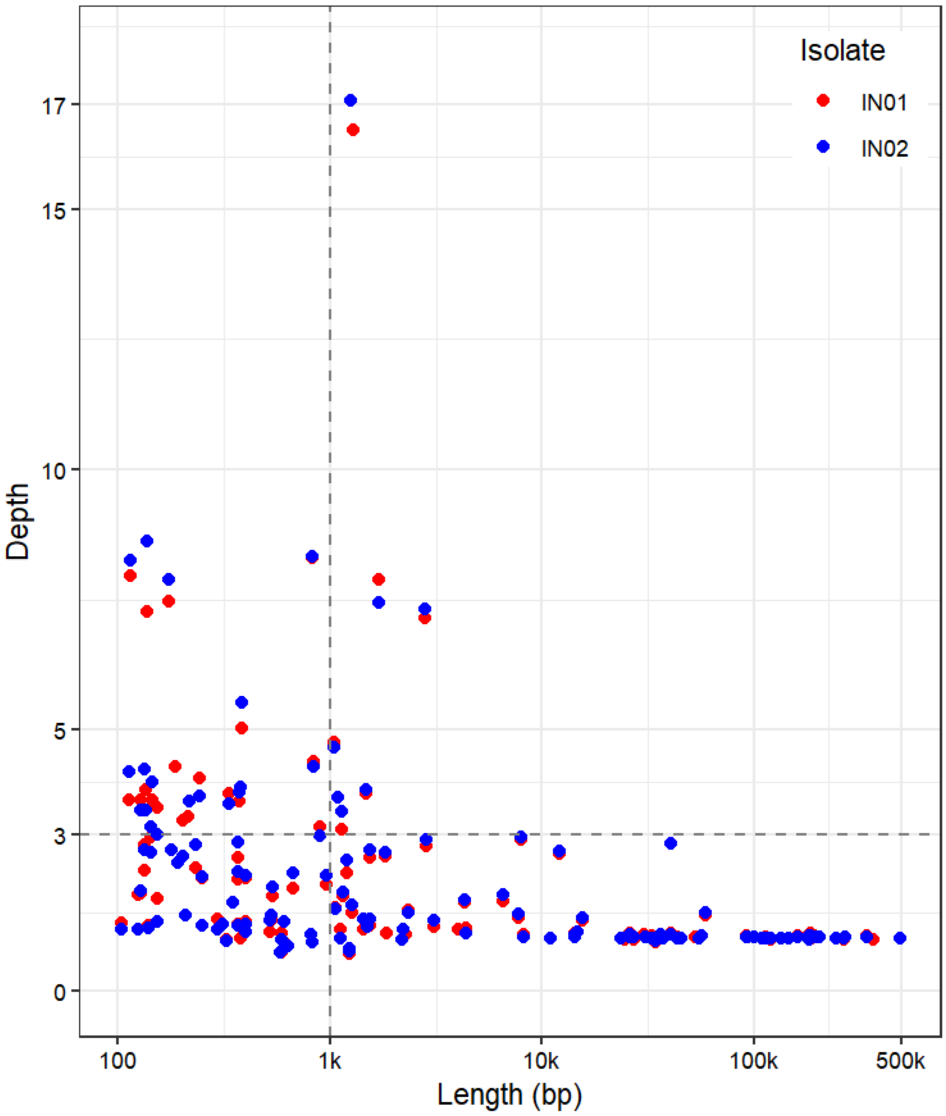

Fig. S5. Contig lengths versus *SPAdes* assembly depths for genome assemblies IN01 and IN02.
