# Supplementary material for: Alterations in chromosomal genes nfsA, nfsB, and ribE are associated with nitrofurantoin resistance in Escherichia coli from the United Kingdom
Source: Microb Genom. 2021 Dec 3;7(12):000702. doi: 10.1099/mgen.0.000702 (PMC8767348; doi:10.1099/mgen.0.000702)
Supplement: Supplementary material 2 [file mgen-7-0702-s002.pdf]

Alterations in Chromosomal Genes *nfsA*, *nfsB*, and *ribE* Are Associated with Nitrofurantoin Resistance in *Escherichia coli* from the UK

Supplementary Tables S3–S5

Yu Wan, Ewurabena Mills, Rhoda C.Y. Leung, Ana Vieira, Xiangyun Zhi, Nicholas J. Croucher, Neil Woodford, Elita Jauneikaite, Matthew J. Ellington, and Shiranee Sriskandan

August 2021

We list known missense mutations in NfsA, NfsB, and RibE that were exclusively identified in *E. coli* isolates showing reduced nitrofurantoin susceptibility. Particularly, we only consider experimentally confirmed (such as using stepwise mutations) causative mutations and those having a deleterious impact on protein structure or translation by prediction. Absence of these mutations in known nitrofurantoin-susceptible *E. coli* was confirmed by aligning wildtype protein sequences from the NITREc database with MEGA X.

Table S3. NfsA missense mutations associated with reduced nitrofurantoin susceptibility in *E. coli*.

Pos.	Ref.	Alt.	Ref. accession	Alt. accession	Confidence	Citation
1	M	I	NG	NA	Possible reduced translation	(1)
11	H	Y	WP_000189141.1[+]	QEL51189.1	PROVEAN prediction	(2)
11	H	Y	AAC73938.1	WP_119187568.1[$]	PROVEAN prediction	(3)
11	H	L	AAC73938.1[*]	NA	Experimentally confirmed	(4)
15	R	C	NG	NA	Experimentally confirmed	(1,5)
33	S	R	AAC73938.1	WP_094323839.1[$]	PROVEAN prediction	(1,3)
33	S	N	WP_000189165.1[+]	QEL51188.1	PROVEAN prediction	(2)
36	A	V	NG	NA	PROVEAN prediction	(5)
38	S	Y	WP_000189167.1[+]	QEL51184.1	PROVEAN prediction	(2)
67	Q	L	AAC73938.1	NA	PROVEAN prediction	(3)
80	C	R	AAC73938.1	NA	PROVEAN prediction	(3)
89	I	N	AAC73938.1[*]	NA	Experimentally confirmed	(4)
126	G	R	AAC73938.1	WP_023149811.1[$]	PROVEAN prediction	(3)
131	G	D	NG	NA	Previously reported	(1)
133	R	S	AAC73938.1[#]	WP_096948202.1	In active-site pocket	(6)
154	G	E	AAC73938.1	WP_000189151.1[$]	PROVEAN prediction	(3)
158	G	C	NG	NA	PROVEAN prediction	(5)
203	R	L/A	NG	NA	Experimentally confirmed	(1,7)
203	R	C	AAC73938.1[#]	WP_072644388.1	Experimentally confirmed	(1,3,6,7)
**212**	**W**	**R**	WP_000189134.1[+]	QEL51185.1	PROVEAN prediction	(2)

Mutations identified in IN01–09 are highlighted in boldface. Pos: Position; Ref: reference amino acid; Alt: alternative amino acid; Ref. accession: NCBI accession of the reference protein sequence; Alt. accession: NCBI accession of the mutant protein sequence; NG: not given in the cited literature; NA, not available. * Based on the nucleotide sequence offered in Fig. 1 of literature (4), we confirmed that the reference *nfsA* allele in *E. coli* strain K-12 sub-strain AB1157 was

identical to that in K-12 sub-strain MG1655, hence we use the protein sequence (NCBI protein accession: AAC73938.1) from MG1655 for the reference. No exact match of either mutant protein (carrying mutation H11L or I89N) was found in the NCBI protein database for *E. coli* (accessed in August 2020). Two mutations (R15C and Q67P) from Table 2 of literature (4) are not included here as we could not solve their discrepancy to mutations illustrated in Fig. 1 of the same article. [+] Reference sequence was restored from mutants and searched against the NCBI protein database (accessed in August 2020) for perfect matches. [#] This *nfsA* allele is identical to that in K-12 sub-strain MG1655. Hence the accession given by Ho, et al. in their article (6), EIE36436.1, refers to an identical protein referred to by accession AAC73938.1. [$] Identical protein found in the NCBI protein database, accessed in September 2020.

Table S4. NfsB missense mutations associated with reduced nitrofurantoin susceptibility in *E. coli*.

Pos.	Ref.	Alt.	Ref. accession	Alt. accession	Confidence	Citation
1	M	I	NG	NA	Possible reduced translation	(1)
33	L	P	AAC73679.1[*]	NA	Experimentally confirmed	(4)
34	L	R	AAC73679.1[*]	WP_096943843.1	Experimentally confirmed	(4)
37	S	R	AAC73679.1[*]	NA	Experimentally confirmed	(4)
40	S	C	AAC73679.1[*]	NA	Experimentally confirmed	(4)
44	Q	L	NG	NA	Experimentally confirmed	(1)
44	Q	P	AAC73679.1[*]	NA	Experimentally confirmed	(4)
44	Q	H	AAC73679.1	EFN8391251.1[$]	PROVEAN prediction	(3)
45	P	S	WP_000351450.1[+]	QEL51196.1	PROVEAN prediction	(2)
45	P	Q	AAC73679.1[*]	NA	Experimentally confirmed	(4)
71	N	K	AAC73679.1[*]	NA	Experimentally confirmed	(4)
84	F	S	WP_000351450.1[+]	QEL51192.1	PROVEAN prediction	(2)
84	F	S	AAC73679.1	NA	PROVEAN prediction	(3)
93	V	G	NG	NA	Experimentally confirmed	(1)
105	D	Y	AAC73679.1[*]	NA	Experimentally confirmed	(4)
107	R	H	NG	NA	Experimentally confirmed	(1)
107	R	C	AAC73679.1	WP_096996177.1[$]	PROVEAN prediction	(3)
138	W	C	NG	NA	Experimentally confirmed	(1)
148	G	S	AAC73679.1[*]	NA	Experimentally confirmed	(4)
165	E	A	NG	NA	Experimentally confirmed	(1)
166	G	D	NG	NA	Experimentally confirmed	(1)
**192**	**G**	**D**/A	NG	NA	Experimentally confirmed	(1)
192	G	S	AAC73679.1	NA	PROVEAN prediction	(3)
207	R	H	AAC73679.1[*]	NA	Experimentally confirmed	(3,4)

Mutations identified in IN01–09 are highlighted in boldface. Pos: Position; Ref: reference amino acid; Alt: alternative amino acid; Ref. accession: NCBI accession of the reference protein sequence; Alt. accession: NCBI accession of the mutant protein sequence; NG: not given in the cited literature; NA, not available. [*] Based on the nucleotide sequence offered in Fig. 2 of literature (4), we confirmed that the reference *nfsB* allele in *E. coli* strain K-12 sub-strain AB1157 was identical to that in K-12 sub-strain MG1655, hence we use the protein sequence (NCBI protein

accession: AAC73679.1) from MG1655 for the reference. An exact match to the protein harbouring mutation L34R was identified in the NCBI protein database for *E. coli*, although no exact match to the mutant DNA sequence was found in GenBank. Four mutations (S39C, V83D, Y183D, and V187E) from Table 2 of literature (4) are not included here as we could not solve their discrepancy to mutations illustrated in Fig. 2 of the same article. [+] A reference protein sequence was restored from both mutants, and it completely matched to WP_000351450.1 in the NCBI protein database. Database searches were performed in August 2020. [$] Identical protein found in the NCBI protein database, accessed in September 2020.

Table S5. RibE missense mutations associated with reduced nitrofurantoin susceptibility in *E. coli*.

Pos.	Ref.	Alt.	Ref. accession	Alt. accession	Confidence	Citation
55	P	H	WP_001021161.1	NA	PROVEAN prediction	(3)
85	G	C	WP_001021161.1[*]	QEL51191.1	PROVEAN prediction	(2)

Pos: Position; Ref: reference amino acid; Alt: alternative amino acid; Ref. accession: NCBI accession of the reference protein sequence; Alt. accession: NCBI accession of the mutant protein sequence; NG: not given by the cited literature; NA, not available. [*] Reference sequence was restored from the mutant and searched against the NCBI protein database (accessed in August 2020) for perfect matches.

References

1. Sandegren L, Lindqvist A, Kahlmeter G, Andersson DI. Nitrofurantoin resistance mechanism and fitness cost in *Escherichia coli*. J Antimicrob Chemother [Internet]. 2008;62(3):495–503. Available from: http://jac.oxfordjournals.org/content/62/3/495.abstract
2. Mottaghizadeh F, Mohajjel Shoja H, Haeili M, Darban-Sarokhalil D. Molecular epidemiology and nitrofurantoin resistance determinants from nitrofurantoin non-susceptible *Escherichia coli* isolated from urinary tract infections. J Glob Antimicrob Resist [Internet]. 2020;21:335–9. Available from: http://www.sciencedirect.com/science/article/pii/S2213716519302565
3. Sorlozano-Puerto A, Lopez-Machado I, Albertuz-Crespo M, Martinez-Gonzalez LJ, Gutierrez-Fernandez J. Characterization of Fosfomycin and Nitrofurantoin Resistance Mechanisms in *Escherichia coli* Isolated in Clinical Urine Samples. Antibiotics [Internet]. 2020;9(9):534. Available from: https://doi.org/10.3390/antibiotics9090534
4. Whiteway J, Koziarz P, Veall J, Sandhu N, Kumar P, Hoecher B, et al. Oxygen-Insensitive Nitroreductases: Analysis of the Roles of *nfsA* and *nfsB* in Development of Resistance to 5-Nitrofuran Derivatives in *Escherichia coli*. J Bacteriol [Internet]. 1998;180(21):5529–39. Available from: http://jb.asm.org/content/180/21/5529.abstract
5. Zhang X, Zhang Y, Wang F, Wang C, Chen L, Liu H, et al. Unravelling mechanisms of nitrofurantoin resistance and epidemiological characteristics among *Escherichia coli* clinical isolates. Int J Antimicrob Agents [Internet]. 2018;52(2):226–32. Available from: http://www.sciencedirect.com/science/article/pii/S0924857918301304
6. Ho P-L, Ng K-Y, Lo W-U, Law PY, Lai EL-Y, Wang Y, et al. Plasmid-Mediated OqxAB Is an Important Mechanism for Nitrofurantoin Resistance in *Escherichia coli*. Antimicrob Agents Chemother [Internet]. 2015 Nov 9;60(1):537–43. Available from: https://pubmed.ncbi.nlm.nih.gov/26552976

7. Kobori T, Sasaki H, Lee WC, Zenno S, Saigo K, Murphy MEP, et al. Structure and site-directed mutagenesis of a flavoprotein from *Escherichia coli* that reduces nitrocompounds: alteration of pyridine nucleotide binding by a single amino acid substitution. J Biol Chem [Internet]. 2001 Jan 26;276(4):2816–23. Available from: http://www.jbc.org/content/276/4/2816.abstract
