# Supplementary material for: Alterations in chromosomal genes nfsA, nfsB, and ribE are associated with nitrofurantoin resistance in Escherichia coli from the United Kingdom
Source: Microb Genom. 2021 Dec 3;7(12):000702. doi: 10.1099/mgen.0.000702 (PMC8767348; doi:10.1099/mgen.0.000702)
Supplement: Supplementary material 3 [file mgen-7-0702-s003.pdf]

# Alterations in Chromosomal Genes *nfsA*, *nfsB*, and *ribE* Are Associated with Nitrofurantoin Resistance in *Escherichia coli* from the UK

Supplementary Table S8

Yu Wan, Ewurabena Mills, Rhoda C.Y. Leung, Ana Vieira, Xiangyun Zhi, Nicholas J. Croucher, Neil Woodford, Elita Jauneikaite, Matthew J. Ellington, and Shiranee Sriskandan

September 2021

**Prediction and validation using published genomic and phenotypic data**

Based on genetic alterations identified using the same BLAST-based method, the prediction was also applied to NCBI isolates that showed nitrofurantoin MICs ≥ 64 mg/L.

For the 27 out of 217 NCBI isolates showing reduced nitrofurantoin susceptibility (MICs ≥ 64 mg/L), our predictions matched the phenotypes of 23 (85%) isolates (Table S7). No *oqxA* or *oqxB* was detected in any of these 27 isolates, and the same "wildtype" *ribE* allele (carried by the nitrofurantoin-susceptible *E. coli* strain ATCC25922) was found in every isolate. Of these 23 isolates, those had mutations in *nfsA* or *nfsB* had MICs ≤ 64 mg/L, and those had mutations in both genes had MICs > 64mg/L, which is consistent with the MICs of the HPRU isolates in Table 4. As for the four exceptions, three isolates that might carry a dysfunctional *nfsA* (URM94, BWH199, and BWH475) had a higher nitrofurantoin MIC (128 mg/L) than expected (≤64 mg/L), which might reflect the ordinary two-dilution fluctuation in the MICs determined for the same isolate when an antimicrobial susceptibility test is repeated (1). Nevertheless, this might not be the case for isolate UCI65, for which the prediction (≤32 mg/L) remarkably differed from the reported MIC (128 mg/L). Since the *nfsA*, *nfsB*, and *ribE* alleles of this isolate were the same as its most closely related isolate BWH453 (Fig. 1), which differed from UCI65 by 796 SNPs. These discrepancies might result from novel resistance mechanisms or reflect incorrect records that require experimental confirmations.

**Table S8. Mutations and nitrofurantoin susceptibility of the 27 NCBI *E. coli* isolates.**

| Isolate | *nfsA*/NfsA (PROVEAN score) | *nfsB*/NfsB | *r* | Susceptibility | | MIC (mg/L) |
|---|---|---|---|---|---|---|
| | | | | Predicted | Observed | |
| URMC09 | Nonsense mutation | Wildtype | 1.0 | S(I) | S(I) | 64 |
| URMC13 | Nonsense mutation | Wildtype | 1.0 | S(I) | S(I) | 64 |
| URMC51 | Nonsense mutation | Wildtype | 1.0 | S(I) | S(I) | 64 |
| URMC52 | Nonsense mutation | Wildtype | 1.0 | S(I) | S(I) | 64 |
| URMC53 | Nonsense mutation | Wildtype | 1.0 | S(I) | S(I) | 64 |
| URMC83 | Nonsense mutation | Wildtype | 1.0 | S(I) | S(I) | 64 |
| URMC85 | Nonsense mutation | Wildtype | 1.0 | S(I) | S(I) | 64 |
| URMC92 | Nonsense mutation | Wildtype | 1.0 | S(I) | S(I) | 64 |
| URMC95 | Nonsense mutation | Wildtype | 1.0 | S(I) | S(I) | 64 |
| URMC94 | Nonsense mutation | Wildtype | 1.0 | S(I) | R | 128 |
| URMC97 | Nonsense mutation | M1R (start-codon loss) | 2.0 | R | R | 128 |
| UCI65 | Wildtype | Wildtype | 0 | S | R | 128 |
| BWH199 | **Y199D** (-9.374) | Wildtype | 0.1 | S or S(I) | R | 128 |

| | | | | | | |
|---|---|---|---|---|---|---|
| BWH403 | 563^564insACTGG…GAGCA | Wildtype | 1.0 | S(I) | S(I) | 64 |
| BWH407 | 563^564insACTGG…GAGCA | Wildtype | 1.0 | S(I) | S(I) | 64 |
| URMC31 | Nonsense mutation | Wildtype | 1.0 | S(I) | S(I) | 64 |
| URMC68 | 102:105delCCGT | Wildtype | 1.0 | S(I) | S(I) | 64 |
| URMC91 | Nonsense mutation | Nonsense mutation | 2.0 | R | R | 128 |
| BWH475 | **L157F** (-3.290), **L232S** (-5.149) | Wildtype | 0.1 | S or S(I) | R | 128 |
| BWH259 | **G125D** (-6.693) | Wildtype | 0.1 | S or S(I) | S(I) | 64 |
| URMC34 | **R203S** (-5.408) | Wildtype | 1.0 | S(I) | S(I) | 64 |
| ST540a | Δ*nfsA* (2 fragments) | Δ*nfsB* (2 fragments) | 2.0 | R | R | 256 |
| ST540an | Nonsense mutation | Wildtype | 1.0 | S(I) | S(I) | 64 |
| BWH237 | N54K (1.445) | Wildtype | 0.1 | S or S(I) | S(I) | 64 |
| UCI57 | Wildtype | Q137E (-0.539) | 0.1 | S or S(I) | S(I) | 64 |
| UCI58 | Wildtype | Q137E (-0.539) | 0.1 | S or S(I) | S(I) | 64 |
| ST2747a | Nonsense mutation | Nonsense mutation | 2.0 | R | R | 128 |

IS*1*-mediated interruptions of both *nfsA* and *nfsB* in ST540a, nonsense mutations in *nfsA* of ST540an, and nonsense mutations in both *nfsA* and *nfsB* have been reported by Vervoort et al (2). Abbreviations: ins, insertion, with its location indicated by positions of left and right flanking nucleotides (left ^ right); del, deletion or truncation, with its start and end nucleotide positions noted on the left side. Deleterious amino acid substitutions (PROVEAN score ≤-2.5) are highlighted in boldface.

## References


1. Reynolds R, Hope R, Warner M, MacGowan AP, Livermore DM, Ellington MJ, et al. Lack of upward creep of glycopeptide MICs for methicillin-resistant *Staphylococcus aureus* (MRSA) isolated in the UK and Ireland 2001–07. J Antimicrob Chemother [Internet]. 2012 Dec 1;67(12):2912–8. Available from: https://doi.org/10.1093/jac/dks324
2. Vervoort J, Xavier BB, Stewardson A, Coenen S, Godycki-Cwirko M, Adriaenssens N, et al. An *In Vitro* Deletion in *ribE* Encoding Lumazine Synthase Contributes to Nitrofurantoin Resistance in *Escherichia coli*. Antimicrob Agents Chemother [Internet]. 2014 Dec 1;58(12):7225 LP – 7233. Available from: http://aac.asm.org/content/58/12/7225.abstract
